# Global randomized controlled trial of knowledge translation of children’s environmental health

**DOI:** 10.3389/fpubh.2025.1502006

**Published:** 2025-03-20

**Authors:** Rivka Green, Christine Till, Jana El-Sabbagh, Allya DaCosta, Erica Phipps, Carly V. Goodman, David B. Flora, Bruce Lanphear

**Affiliations:** ^1^The Hospital for Sick Children, Toronto, ON, Canada; ^2^Faculty of Health, York University, Toronto, ON, Canada; ^3^Canadian Partnership for Children’s Health and Environment (CPCHE), Ottawa, ON, Canada; ^4^Faculty of Health Sciences, Simon Fraser University, Vancouver, BC, Canada

**Keywords:** brain development, developmental neurotoxicology, knowledge translation, prevention, health literacy

## Abstract

**Objectives:**

Toxic chemicals can harm children’s brain development, but the public’s understanding of these harmful impacts is largely unknown. People’s knowledge of toxic chemicals and their awareness of how to reduce children’s exposure was examined. This study also assessed whether a video was efficacious in increasing knowledge about toxic chemicals and brain development and encouraging behavioral change to reduce exposure to toxic chemicals.

**Methods:**

15,594 participants of child-bearing age (18–45 years old) from five countries (Canada, United States, United Kingdom, India, and Australia) were surveyed via CloudResearch’s Prime Panels®. After completing a baseline survey, Prevention of Toxic Chemicals in the Environment for Children Tool (PRoTECT), participants were randomly assigned to watch a knowledge translation video (experimental group) or serve as a control group. Next, participants were asked about barriers and intentions to reduce exposure to toxic chemicals. After 6 weeks, a subset (*n* = 4,842) of participants were surveyed with PRoTECT and asked whether they modified behaviors to reduce exposure to toxic chemicals or plan to speak to their healthcare provider (HCP) about toxic chemicals.

**Results:**

Participants expressed strong preferences for lowering exposures and preventing disabilities. Participants who knew more about the impact of toxic chemicals on children’s health were more likely to prefer investing in prevention and reducing their exposures. Participants who viewed the video showed significantly greater changes in PRoTECT scores. At the 6-week follow-up, no differences in behavioral changes were observed by group assignment, but two-thirds of all participants reported making changes to reduce their exposures and half intended to speak with their HCP.

**Conclusion:**

There were significant differences in knowledge and preferences by group assignment, but systemic barriers, such as cost of non-toxic products and difficulty determining how and where to buy them, hindered people from making changes to reduce their exposures to toxic chemicals.

## 1 Introduction

Over the past 20 years, the prevalence of neurodevelopmental disorders (NDDs) in the United States (U.S.), such as attention deficit hyperactivity disorder (ADHD) and autism spectrum disorder (ASD), has risen from 13 to 18% (1), indicating a need to identify modifiable risk factors and develop policies to prevent NDDs in children. Toxic chemicals, like lead and pesticides, increase the risk of NDDs (2), but legislation to reduce widespread exposure to toxic chemicals and pollutants is inadequate (3). Information about the harmful impact of toxic chemicals can galvanize people to reduce their family’s exposure to toxic chemicals and accelerate the adoption of legislation to protect children from these chemicals (4).

Little is known about parents’ knowledge of toxic chemicals or their ability to reduce their children’s exposure (5–9). If parents are aware of the risks, they may be more likely to make changes to reduce their children’s exposure to toxic chemicals (4). In 2015 and 2018, Healthy Babies Bright Futures surveyed over 1,000 adults and found that after sending messages, such as, “studies show that more than 90% of American women of childbearing age have toxic chemicals in their bodies at a level that will increase the risk of brain damage and loss of intelligence in their babies,” most respondents said chemicals were a serious threat. However, the study did not examine whether participants attempted to reduce their exposure. In a 2021 Canadian survey of approximately 2000 women of reproductive age, more than 90% agreed or strongly agreed that day-to-day exposures can be harmful to children’s health and that pregnant people can reduce their risk by reducing exposure to toxic chemicals (10). However, the study did not assess whether they acquired knowledge by completing the survey or test the effect of an intervention.

Little is known about the efficacy of tools to educate people of childbearing age about toxic chemicals. Two studies conducted interventions with families to investigate their attitudes and behaviors about residential hazards, such as lead and radon (11, 12). These studies indicated that their interventions improved participants’ knowledge of hazards, self-efficacy, and adoption of environmental health (EH) precautions (11, 12) but, they did not assess knowledge of toxic chemicals or families’ preferences for prevention of NDDs.

The current study examined people of childbearing age’s knowledge of toxic chemicals and their knowledge of how to reduce children’s exposure using the Prevention of Toxic Chemicals in the Environment for Children Tool (PRoTECT) questionnaire (13). A second aim evaluated the efficacy of a video, Little Things Matter: The Impact of Toxins on the Developing Brain (14), on increasing people’s knowledge of toxic chemicals’ impact on children’s development, both immediately after watching the video and after a 6-week interval.

## 2 Methods

### 2.1 Study design

15,594 participants were recruited aged 18 to 45 years from Canada, the U.S., the United Kingdom (U.K.), India, and Australia via CloudResearch’s Prime Panels®, an online platform commonly used for behavioral research (15). Before participating in this trial, participants were provided with a detailed consent form outlining the study’s objectives, procedures, potential risks and benefits. Participants were informed that their involvement was voluntary and that they could withdraw from the study at any time without facing any consequences. Prime Panels® recruits online survey participants to ensure that eligibility criteria and data quality standards are met through CloudResearch’s quality control system, SENTRY®. In this study, SENTRY® prevented approximately 50% of participant traffic recruited into the study due to inattentiveness, reCAPTCHA fails, and similar problems. The sample was stratified based on soft census gender (49% male, 51% female) and age quotas (18–22 years: 15–24%; 23–35 years: 45–51%; and 36–45 years: 25–39%), with quotas adjusted based on country. Stratification was used to survey an international sample, obtain high-quality data, and ensure the sample was representative of the population in terms of race, gender, and socioeconomic status. Eligible participants anonymously completed the study through an online survey platform, Qualtrics, and received a small monetary incentive prior to completion.

**Figure 1:**
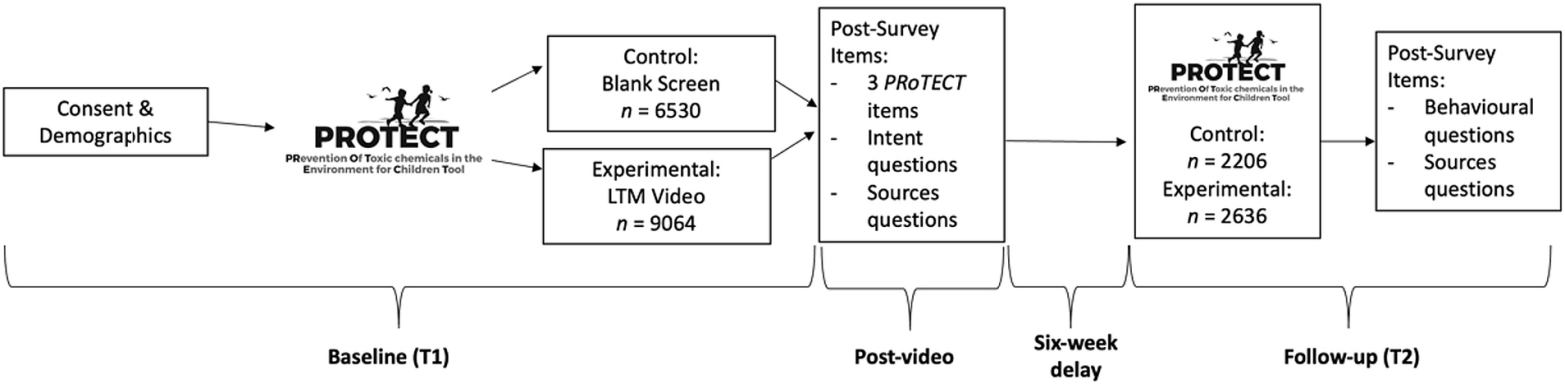
Study timeline.

Participants completed a brief demographic questionnaire prior to the administration of the PRoTECT questionnaire. The PRoTECT questionnaire was used to measure baseline knowledge and preferences about toxic chemicals and their impact on children’s brain development. Next, participants were randomly assigned to the experimental group or control group using a built-in randomizer in the survey tool, to quantify the efficacy of the Little Things Matter: The Impact of Toxins on the Developing Brain video (Appendix A) on knowledge, preferences, and changing behaviors to reduce exposure to toxic chemicals. The experimental group (*n* = 9,064) watched the video whereas the control group (*n* = 6,530) briefly viewed a blank screen between baseline and post-survey questions until they clicked the next page button to advance; participants in the experimental group were oversampled to account for potential dropout during the 7-min video. All participants were blinded to their assignment as the purpose of the study or details about the video were not disclosed. All participants completed post-survey questions, including three questions from PRoTECT and questions about their intention to reduce exposure to toxic chemicals through their choices of household, food, and personal care products (Appendix B). Six weeks after the baseline survey, all participants were contacted to complete the follow-up survey. However, due to low retention rates common with online survey platforms like CloudResearch, only a subset of participants (31.1%) completed the follow-up survey (Figure 1). This retention rate, which is typically around 15 to 20%, is consistent with other online survey data collection sites (15).

Data collection occurred between October and December 2021. This study was approved by York University’s research ethics board.

### 2.2 Tools

#### 2.2.1 Prevention of toxic chemicals in the environment for children tool (PRoTECT)

Our team developed and validated the PRoTECT questionnaire to examine the public’s knowledge and concerns about toxic chemicals and children’s brain development, as well as their preferences towards the prevention of NDDs (13, 16). Questions on PRoTECT were refined through focus groups (*n* = 46) to ensure clarity and relevance, followed by expert evaluation of content validity (*n* = 17) (13). The questionnaire was then administered to 190 participants of child-bearing age, and exploratory factor analysis confirmed a robust four-factor structure (RMSR = 0.05), with 16 of 18 items demonstrating strong content validity and factor loadings exceeding 0.40. PRoTECT showed good interpretability and acceptability, identifying three themes: (1) concern about exposure and preferences to lower exposure; (2) knowledge and perception of the regulation of toxic chemicals by government and industry; and (3) knowledge of developmental neurotoxicity (Supplementary Table S1). The final PRoTECT questionnaire consists of 17 questions with a five-point Likert scale ranging from strongly agree to strongly disagree or vice-versa (Appendix D). Scores for each theme were calculated by averaging the scores for items corresponding to each factor.

### 2.3 Knowledge translation video

The Impact of Toxins on the Developing Brain is a 7-min video highlighting the widespread nature of toxic chemical exposures and how low-level exposure to toxic chemicals can diminish children’s intellectual abilities (2). The video concludes with a list of ways to reduce exposure to toxic chemicals. To assess participants’ attention and comprehension throughout the video, after watching the video, participants were asked to select whether the video was related to (a) the history of the chemical industry, (b) the impact of toxic chemicals on brain development (correct answer), (c) growth of the U.S. population over the past 20 years, or (d) trends in intellectual quotient (IQ) scores over time. Respondents who did not answer correctly were instructed to exit the study prior to the survey.

### 2.4 Post-video questions

Post-video questions were administered to examine the efficacy of the video (Appendix B). Participants were also asked to complete three questions from PRoTECT gathered from the three themes [(1) preferences to lower exposure and increase prevention, (2) attitudes towards regulations of toxic chemicals, (3) knowledge of developmental neurotoxicity] to determine if the video affected their responses immediately after viewing the video.

Six weeks after the baseline survey, participants (*N* = 2,206 for the control group; *N* = 2,636 for the experimental group) were surveyed about whether they modified their behaviors to reduce exposure or planned to speak to their healthcare provider (HCP) (Appendix C).

### 2.5 Analyses

To determine if viewing the video influenced participants’ knowledge and preferences, responses for higher scores (e.g., agree and strongly agree) were aggregated for each question to compare percent agreement by group assignment. For theme 2, which assesses attitudes and perceptions of the regulation of toxic chemicals by government or industry, a more desirable response (i.e., wanting the government to implement more policies to reduce exposure or increase prevention) was consistent with “strongly disagree” (e.g., “Most governments spend about the same amount to prevent developmental conditions as they spend to treat these conditions”). As such, “strongly disagree” was coded as a 5 (a higher score), and strongly agree was coded as a 1. Therefore, higher scores reflect a stronger preference for prevention (i.e., implementing more policies to reduce exposure) and the perception that industry or government should do more to protect children from toxic chemicals. This was done to maintain consistency with scoring structures in themes 1 and 3. In supplemental analyses, mean scores [and standard deviations (SD)] at baseline (pre-video) and at the 6-week follow-up (post-video) were compared between the experimental and control groups to explore whether the video influenced intent, reported behavioral changes and perceived barriers.

Significance testing was also performed to assess the observed changes. Normality testing was first conducted on pre-post difference scores for both groups using the Anderson-darling test. We used non-parametric tests were since scores were not normally distributed in either the experimental group (*A* = 12.013, *p* < 0.001) or the control group (*A* = 7.55, *p* < 0.001). Within-group differences between pre- and post-test scores were assessed using Wilcoxon signed-rank tests for both the experimental and control groups. Between-group differences in pre-post changes were evaluated using a Mann Whitney *U* test. All statistical analyses were conducted using *R* statistical software (version 2024.12.0 + 467). In such a large dataset, even small or non-meaningful differences could produce statistically significant *p*-values, potentially overemphasizing negligible effects (17, 18). For this reason, the results were interpreted primarily using mean scores and SD to better reflect practical and meaningful differences.

## 3 Results

In total, 15,594 participants completed the baseline survey (Table 1). Within the experimental group participants, 75.3% (*n* = 6,827) watched the entire video and only 4.1% (*n* = 281) failed the attention check, leaving 72.2% (*n* = 6,546) participants in the experimental group for post-survey questions.

**Table 1:** Demographic characteristics of participants at baseline by group status.

	Baseline	
	Control group	Experimental group
*n*	6,530	9,064
Gender [frequency (%)]		
Female	3,268 (50.0)	4,861 (53.6)
Male	3,202 (49.0)	4,083 (45.1)
Non-Binary	45 (0.7)	83 (0.9)
Not reported	15 (0.2)	37 (0.4)
Country [frequency (%)]		
Canada	1,100 (16.8)	1729 (19.1)
Australia	1,081 (16.5)	1,594 (17.6)
India	1,294 (19.8)	1892 (20.9)
United Kingdom	1,154 (17.7)	1,630 (18.0)
United States	1901 (29.1)	2,219 (24.5)
Ethnic groups [frequency (%)]		
White	3,416 (52.3)	4,578 (50.5)
Black	481 (7.4)	557 (6.1)
South Asian	1,231 (18.9)	1821 (20.1)
Other	1,402 (21.4)	2,108 (23.3)
Location [frequency (%)]		
Major city	2,342 (35.9)	3,294 (36.3)
Suburban edges	1982 (30.4)	2,661 (29.4)
Major town	667 (10.2)	1,004 (11.1)
Small town	1,303 (19.9)	1757 (19.4)
Remote	176 (2.7)	260 (2.8)
Not reported	60 (0.9)	88 (1.0)
Mean (SD) years of age	32.22 (8.2)	31.65 (8.1)
Level of education [frequency (%)]		
High school or less	1,612 (24.7)	2,125 (23.4)
Some college or university, no degree/diploma	1,085 (16.6)	1,461 (16.1)
Bachelor’s degree or diploma	2,643 (40.5)	3,685 (40.7)
Master’s, doctorate or professional degree	1,115 (17.1)	1,671 (18.4)
Not reported	75 (1.1)	122 (1.3)
Employment [frequency (%)]		
Full-time	3,416 (52.3)	4,596 (50.7)
Part-time	945 (14.4)	1,377 (15.2)
Other (Retired, Student, Self-Employed, Unemployed, Unknown, prefer not to disclose)	1970 (30.2)	2,849 (31.43)
Not reported	199 (3.0)	242 (2.7)
Political leaning^*^		
Mean (SD), Median	5.04 (2.4), 5	4.94 (2.4), 5
*Parental questions*		
Marital status [frequency (%)]		
Single	3,348 (51.3)	4,932 (54.4)
Married / Common law	2,877 (44.1)	3,731 (41.2)
Separated / Divorced or widowed	305 (4.6)	401 (4.4)
Children [frequency (%)]		
Yes, I have children	2,941 (45.0)	3,796 (41.9)
No, I do not have children	3,589 (55.0)	5,268 (58.1)
Pregnancy status [frequency (%)]	3,268	4,861
Pregnant	350 (10.7)	405 (8.3)
Non-pregnant	2,572 (78.7)	3,371 (69.3)
Not Reported	346 (10.6)	1,085 (22.3)
Identified developmental conditions (Participants’ children) [frequency (%)]	2,941	3,796
No	2,303 (78.3)	2,968 (78.2)
Yes	504 (17.1)	665 (17.5)
I do not know	108 (3.7)	127 (3.3)
Not reported	26 (0.9)	36 (0.9)
Identified developmental conditions (Parents) [frequency (%)]	2,941	3,796
No	2,502 (85.1)	3,234 (85.2)
Yes	363 (12.3)	470 (12.4)
I do not know	69 (2.3)	84 (2.2)
Not reported	7 (0.3)	8 (0.2)

^*^ Question: In politics, people sometimes talk about the ‘left’ and the ‘right’. On a scale where ‘0’ means left and ‘10’ means right, where would you place yourself on the scale?

### 3.1 Aim 1: assessing people’s knowledge of toxic chemicals and their awareness on how to reduce children’s exposure

Overall, 67.1% of respondents agreed that environmental chemicals increased the risk for NDDs and 79.7% of participants reported a preference for reducing exposure to toxic chemicals and increasing prevention (Table 2). In contrast, only 32.7% of participants agreed industry and government were doing enough to regulate toxic chemicals. Responses across individual items are provided in Supplementary Table S2.

**Table 2:** Percentages for responses on questions per each theme on PRoTECT.

Content	Strongly disagree	Disagree	Neither agree nor disagree	Agree	Strongly agree	Higher response (agree or strongly agree)
*Theme 1: Do respondents prefer to lower exposure and increase prevention?*	1.3	3.1	15.9	32.6	47.2	79.7
*Theme 2: Is industry and government doing enough to protect children from toxic chemicals?^a^*	17.2	27.6	22.5	20.3	12.3	32.7
*Theme 3: Do environmental chemicals increase risk for developmental neurotoxicity?*	2.5	5.4	25.0	33.6	33.5	67.1

a Theme 2 is reverse-coded, such that 1 is strongly agree and 5 is strongly disagree.

### 3.2 Responses on the PRoTECT survey by demographic characteristics

Participants from India were more likely to agree or strongly agree with a preference for lowering exposure to toxic chemicals and increasing prevention, compared with participants from other countries (Supplementary Table S3). In particular, 67.7% of Indian participants strongly agreed that they wanted to learn more about how to reduce children’s exposure to toxic chemicals, compared with 28–39% of participants from other countries. Higher levels of education were also observed to be associated with higher preference to lower exposure and increase prevention. No substantial differences in mean scores were observed based on gender, pregnancy status, age, or having a child with a neurodevelopmental disorder.

Survey responses indicated that U.S. participants were least likely to trust industry regulations of toxic chemicals and more likely to agree that their government was doing enough to regulate chemicals, compared with participants from other countries (Supplementary Table S3). In addition, 38.1% of U.S. participants strongly disagreed that all parents have equal opportunities to protect their children from toxic chemicals, compared with 24% of Canadian participants, 19% of participants from the U.K., 17.8% of Australian participants, and 9.2% of Indian participants. Females also tended to have slightly higher scores (Supplementary Table S3). Modest differences based on parental and pregnancy status were also observed.

Results indicated that knowledge of developmental neurotoxicity differed by country. These differences were consistent with preferences to lower exposure and increase prevention, such that participants from India had higher scores than participants from other countries (Supplementary Table S3). In particular, 65% of participants from India strongly agreed that gestational exposures to toxic chemicals can increase a child’s risk of having a developmental condition (item 8), compared with 52.3% of participants from the U.S., 43.8% of participants from Canada, 37.9% of participants from Australia, and 30.9% of participants from the U.K.

The percentage of participants with the highest level of education had greater knowledge of developmental neurotoxicity than those with lower levels of education. For example, 46.3% of participants with the highest education level strongly agreed that reducing exposure to toxic chemicals during pregnancy and in early childhood can help lower a child’s risk of developing a condition like ADHD or ASD (item 5), compared to 36.3% of participants with a bachelor’s degree or diploma, 31.7% with some college or university, and 28.4% with a high school degree or less. In contrast to subscale two, males had slightly higher scores than females indicating greater knowledge of toxic chemicals and developmental neurotoxicity (Supplementary Table S3). Furthermore, pregnant women had slightly higher scores compared to non-pregnant women (Supplementary Table S3).

### 3.3 Aim 2: assessing the efficacy of a video on increasing people’s knowledge of toxic chemicals’ impact on children’s development

#### 3.3.1 Differences in responses on the PRoTECT survey immediately following video

**Figure 2:**
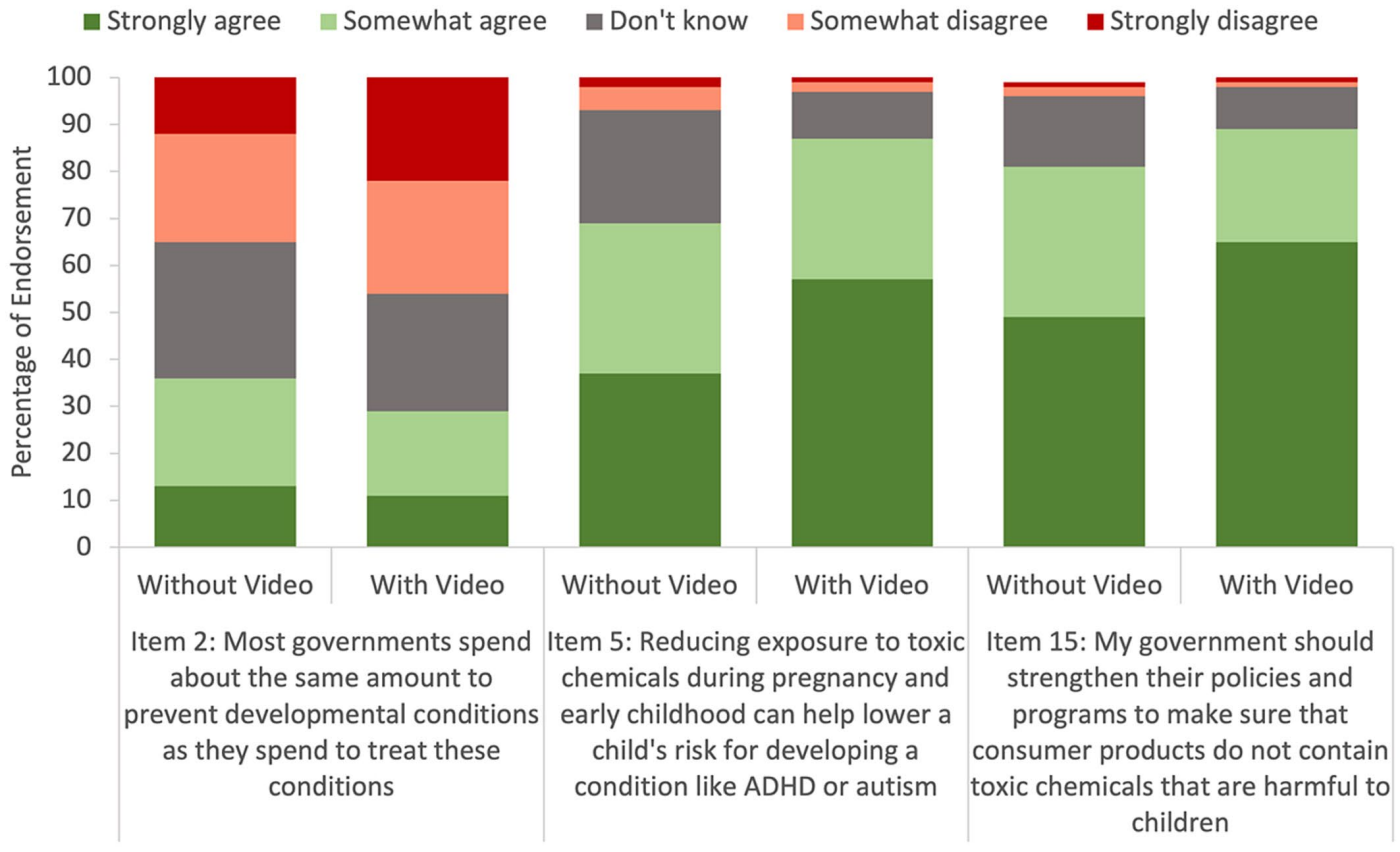
Differences on responses to selected PRoTECT questions by theme immediately after the intervention by group status.

Immediately after watching the video, large differences were observed by group assignment in knowledge of the hazards of toxic chemicals on brain development, attitudes toward government investment in prevention, and preferences for more governmental spending in prevention (Figure 2). A larger percentage of participants who watched the video somewhat disagreed or strongly disagreed that governments spend as much on prevention of developmental conditions as on treatment (Figure 2). Participants in the experimental group relative to the control group were also more likely to somewhat agree or strongly agree that reducing exposure to toxic chemicals can lower the risk of developmental conditions like ADHD or autism and that government policies should be strengthened to ensure consumer products are free from harmful toxic chemicals (Figure 2).

Experimental group participants were also more confident in their ability to explain the role of toxic chemicals on brain development, with 75.2% indicating they would feel somewhat or very likely comfortable doing so, compared to 61.3% in the control group. In contrast, there were no group differences in the intent to change food consumption patterns or personal-care products (Table 3).

**Table 3:** Percentages of intentions to reduce exposure to toxic chemicals over the next month at baseline by group status.

Question	Group status	Very unlikely	Somewhat unlikely	Neither likely nor unlikely	Somewhat likely	Very likely	Higher response (somewhat or very likely)
*Would you feel comfortable explaining to a friend that toxic chemicals can impact brain development?*	Control	4.3	12.3	22.1	32.8	28.5	61.3
	Experimental	2.3	7.5	15.0	39.8	35.4	75.2
*How likely are you to make any changes to reduce toxic chemicals in your household?*	Control	1.7	4.6	18.1	39.0	36.5	75.5
	Experimental	1.5	3.9	13.0	37.3	44.3	81.6
*How likely are you to make any changes to the foods you buy or eat to reduce your exposure to toxic chemicals?*	Control	1.9	5.8	17.2	37.9	37.2	75.1
	Experimental	2.3	4.8	13.9	36.1	42.9	79.0
*How likely are you to make any changes in your use of personal-care products to reduce your exposure to toxic chemicals?*	Control	1.8	5.1	17.4	38.1	37.6	75.7
	Experimental	1.9	4.8	14.1	36.1	43.1	79.2

Some perceived barriers to reducing exposure to toxic chemicals differed by group assignment (Table 4). Only 13.8% of participants in the experimental group reported they did not know how to reduce toxic chemical exposure, compared to 23.8% of participants in the control group. Only 5% of participants in both groups did not believe that reducing toxic chemicals would make a difference to their health, while more than 20% of participants across groups said that the cost of non-toxic products and not knowing where to purchase them was a barrier.

**Table 4:** Barriers to reduce exposure to toxic chemicals immediately following intervention at baseline by group status.

Barriers	Control group (%) *N* = 9,703	Experimental group (%) *N* = 9,784
I do not know how to reduce toxic chemicals	23.8	13.8
Non-toxic products and food cost too much	21.0	26.1
It is not convenient to shop in stores where less toxic questions are available	15.1	17.1
I do not know where to purchase non-toxic items or food	20.5	20.8
I do not believe it makes a difference to my health/my family’s health	4.1	3.2
I do not have support from my partner or other family member	3.0	3.8
I do not have time	4.5	4.8
Other	0.9	1.2
None of the above apply to my situation	7.3	9.3

Participants could check off as many barriers as applied.

In the experimental group, greater mean increases were observed in subscale scores compared to the control group. The mean change over time by group was greatest for subscale two, with almost three times greater change in the experimental group (control group = 0.11 and experimental group = 0.31), in comparison to subscale one (control group = 0.06 and experimental group = 0.10) and subscale three (control group = 0.12 and experimental group = 0.21). This means that the video induced a greater change in attitudes towards regulations of toxic chemicals (subscale two), with smaller differences in change for preferences to lower exposure and increase prevention (subscale one), as well as knowledge of developmental neurotoxicity (subscale three).

Wilcoxon signed-rank tests revealed significant pre-post changes within both groups. The experimental group showed a higher significant change in scores (*V* = 1,054,450, *p* < 0.001), as compared to the control group (*V* = 890,901, *p* = 0.028). A Mann–Whitney U test further confirmed that the pre-post differences were significantly greater in the experimental group compared to the control group (*W* = 2,997,334, *p* < 0.001). This indicates that the video had a more pronounced effect on participants’ knowledge, attitudes, and preferences for prevention policies in the experimental group compared to the control group.

#### 3.3.2 Differences in responses on the PRoTECT survey after a 6-week interval

There were small differences observed in the characteristics of the participants in the control group and experimental group at the 6-week follow-up (Supplementary Table S4). Participants who completed the 6-week follow-up were more likely to be U.S. residents (43.11% in the control group and 56.34% in the experimental group at the 6-week follow-up, compared to 29.1% in the control group and 24.4% in the experimental group at baseline) and have a higher education level (19.95% with a master’s, doctorate or professional degree in the control group and 19.58% in the experimental group at the 6-week follow-up, compared to 17.1% in the control group and 18.4% in the experimental group at baseline). Additionally, the experimental group included more U.S. participants than the control group, while participants from other countries were relatively evenly distributed between groups.

Compared to the control group, the experimental group participants were more likely to agree that (1) the government is not doing enough to regulate toxic chemical exposure, (2) governments should spend more money to prevent developmental conditions in children, (3) babies and young children are more likely to be harmed by exposure to toxic chemicals, (4) preventing developmental conditions in children is more preferred than treatment, and (5) policies and programs should be strengthened to reduce children’s exposure to toxic chemicals (Table 5).

**Table 5:** Differences in scores at follow-up by group status.

	Control Group *N* = 2,206			Experimental Group *N* = 2,636		
	Baseline percent agree or strongly agree	6-week follow-up percent agree or strongly agree	Percent difference	Baseline percent agree or strongly agree	6-week follow-up percent agree or strongly agree	Percent difference
Theme 1: *Preferences to lower exposure and increase prevention*	47	49.7	2.7	47.3	52.4	5.1
Theme 2: *Attitudes towards regulations of toxic chemicals by government and industry*	32.4	37.6	5.2	32.9	43.8	11
Theme 3: *Knowledge of developmental neurotoxicity*	33.7	37.1	3.4	33.3	41.1	7.7

Experimental group participants were also more likely to agree that: governments do not spend the same to prevent conditions as they do to treat those conditions; parents do not have equal opportunities to protect their children; and the government does not have effective regulations to protect children from toxic chemicals. Participants in the experimental group were also less likely to trust that companies were not including harmful levels of toxic chemicals in their products. Compared with the control group, participants in the experimental group had a slightly larger increase in their knowledge of developmental neurotoxicity and were more likely to agree that exposure to toxic chemicals during pregnancy can increase children’s risk of developing a neurodevelopmental condition; toxic chemicals can be found in blood during pregnancy; and reducing children’s exposure to toxic chemicals can lower their risk of developing a neurodevelopmental condition (Supplementary Table S5).

Participants from the U.S. had ≥5% changes to agreement for all questions (Supplementary Table S5), indicating greater overall agreement for the prevention of toxic chemicals in the environment for children at follow-up. Experimental group participants from the U.S. had changes that were 5% or larger than those of control group participants. Participants from other countries did not have as meaningful changes in scores.

Six weeks after the baseline survey, group differences in the behavioral change questions were also examined (Table 6). No meaningful differences were identified by group assignment, suggesting that both groups made changes to reduce their exposure to toxic chemicals.

**Table 6:** Differences in reduction of exposure at 6-week follow-up by group status.

Question (To what extent have you…)	Group Status	Not at all	Somewhat not	Neutral	To some extent	A great extent	Higher response (some or great extent)
*… Changed your household practices to reduce exposures to toxic chemicals?*	Control	8.5	10.0	22.2	45.9	13.4	59.3
	Experimental	9.3	10.1	19.8	50.8	10.1	60.9
*…Made any changes to the foods you buy or eat to reduce your exposure to toxic chemicals?*	Control	8.7	9.4	18.7	46.3	16.9	63.2
	Experimental	11.0	8.2	16.4	50.2	14.3	64.5
*…Made any changes in your purchase or use of personal-care products to reduce your exposure to toxic chemicals?*	Control	10.7	11.4	24.7	38.4	14.7	53.1
	Experimental	14.1	11.6	22.9	38.2	13.3	51.5

While over 70% of participants in both groups reported being concerned that they or their family may be exposed to toxic chemicals, more than half of participants said that cost, inconvenience, and not knowing where to shop were barriers to using non-toxic products (Table 7). Fewer participants in the experimental group (17.0%) reported not knowing how to reduce toxic chemicals compared to those in the control group (26.6%). Most participants reported they had trouble determining whether a product was non-toxic; participants in the experimental group reported slightly less difficulty determining toxicity (52%) than participants in the control group (56%).

**Table 7:** Barriers to reduce exposure to toxic chemicals at 6-week follow-up by group status.

Examples of barriers	Control group *N* = 1934	Experimental group *N* = 2,297
	% reported	
I do not know how to reduce toxic chemicals	26.6	17.0
Non-toxic products and food cost too much	36.4	40.1
It is not convenient to shop in stores where less toxic questions are available	24.8	27.5
I do not know where to purchase non-toxic items or food	22.5	20.9
I do not believe it makes a difference to my health/my family’s health	4.9	4.3
I do not have support from my partner or other family member	5.4	5.2
I do not have time	9.0	10.3

Participants could endorse as many barriers as applied.

Participants were asked whether they plan to speak with their HCP about toxic chemicals. Results were collapsed across groups because no meaningful differences were observed by group status. Approximately 32% of participants indicated they plan to speak with their HCP, while about 15% said that they have already done so. Results indicated that 45% of participants from India planned to speak to their HCPs, compared with 23–33% from other countries. Similarly, 35% of Indian participants said they have already spoken to their HCPs, compared with fewer than 10% from the other countries. Of the 53% of participants who did not plan to speak to their HCP, about 30% said they had more important issues to discuss, 30% said they did not know what to ask, 23% said that did not think their HCP would have the information, 20% said that their HCP might dismiss their concerns, 17% said they were not sure it was a valid question, and 15% said that they felt they would not have time. Only 5% said that they would not trust their advice.

## 4 Discussion

Toxic chemicals are putting children at a higher risk of developing neurodevelopmental disorders, like ADHD and autism. The societal costs of managing these conditions are substantial (19), yet very little is being done to prevent these conditions. Do people know that there may be ways to contribute to preventing these disorders or recognize the government’s role? Little is known about people’s literacy regarding developmental neurotoxicity, their concerns about toxic chemicals, or their preferences for prevention or treatment. A large sample of participants from five countries were surveyed about their knowledge and preferences regarding toxic chemicals and brain development, and the efficacy of a video was tested for producing changes in knowledge and behavior immediately after its viewing, as well as after a 6-week period. Given the large sample size of this study (over 15,000 participants), descriptive statistics and effect sizes were emphasized rather than *p*-values. Statistical tests often display no difference in their power for large sample sizes, so by focusing on effect sizes and descriptive statistics, we aimed to present findings that highlight practical or scientific significance (17, 18).

At baseline, participants knew little about toxic chemicals, as measured by the PRoTECT questionnaire. Furthermore, 74.3% of participants agreed/strongly agreed they want to learn more about how to reduce children’s exposure to toxic chemicals. Participants also knew little about how toxic chemicals are regulated, with 52.3% of participants agreeing/strongly agreeing that their government has effective regulations to ensure that food and personal care products do not contain harmful levels of toxic chemicals. Informing parents about how the government regulates chemicals may encourage them to advocate for change. In fact, watching the video led to changes in perceptions about governments and industry’s role in protecting people from toxic chemicals. The experimental group also expressed a greater preference in support for prevention efforts regarding toxic chemicals than the control group. The larger shifts in attitudes in the experimental group suggest that the video increased participants’ awareness and preference for governmental action on toxic chemical exposure.

Both groups were observed to have had more knowledge of toxic chemicals in the follow-up survey than at baseline. This suggests that participants made some changes to reduce their exposure, but changes did not vary based on group status. Systemic barriers – like cost and difficulty determining how and where to buy non-toxic products – hindered people’s ability to reduce their exposure to toxic chemicals. Indeed, more participants reported that the cost of non-toxic products was a barrier in the follow-up survey than at baseline. In addition, 55% of all participants said they had trouble finding out if a product was non-toxic. Fewer participants in the experimental group said they did not know how to reduce their exposure to toxic chemicals than the control group. Empowering the public with more knowledge about how to make choices is important but may not be sufficient to overcome systemic barriers or change health-related behaviors (20, 21). These same barriers have been found for adopting a healthier diet (22), preventing childhood injuries (23), and implementing strategies towards disease prevention (21). Population strategies are needed to protect children from toxic chemicals and other hazards; it must be easier for people to make healthier choices for their families.

Educating parents can inadvertently blame them for failing to adopt healthier behaviors in the face of systemic barriers. During focus groups with families, qualitative results indicate that parents often feel responsible for controlling their families’ exposures and blamed that they may have ‘caused’ their child to have a NDD (13). One parent said: “*This makes me sad it’s almost like a blame game. We’re trying to find out if my son has autism currently and some of the questions our doctor asked sound like they are going to blame you for it*” (13). Government agencies and industry are responsible for regulating pollutants and exposure to toxic chemicals. Almost 80% of all respondents and 86% of parents agreed their government should regulate toxic chemicals to prevent developmental disorders in children. Parents recognize that the onus must be on government and industry to make it easier to stay healthy and harder to get sick.

Further, while parents’ advocacy to create change can be empowering, relying on this way to accelerate reform is problematic because most parents are unaware of how the system is failing to protect them until it is too late. Over half of the participants agreed that industry and government are doing enough to regulate toxic chemicals. After watching the video, the largest change in scores was for questions on the regulation of toxic chemicals and government investment in prevention. Participants in the experimental group were more likely to disagree that the government had effective regulations to control toxic chemicals, from 23% in the baseline to 34% in the follow-up. In addition, experimental group participants were more likely to agree that not enough resources were devoted to preventing NDDs.

Despite this, parents’ understanding of the impact of toxic chemicals on children’s development may accelerate the promulgation of protective policies and regulations. Historically, parents have accelerated change, such as Mothers Against Drunk Drivers (MADD) and the water disaster in Flint, Michigan (24, 25). While we await stronger legislation to reduce toxic exposure among pregnant women and children, it is important to continue to find ways to effectively communicate these risks with parents and caregivers without inciting blame and guilt. The PRoTECT questionnaire is a useful tool for assessing knowledge about toxic chemicals and their impact on children’s neurodevelopment. In addition to examining patterns of knowledge, preferences, and concerns, PRoTECT can serve as a prompt to educate people on the risks of toxic chemicals and encourage them to begin to look for more information. In fact, in this study, 60% of people in the control group made between one and three changes to reduce their exposure to toxic chemicals, 55% looked for more information on the subject, and about a third had a conversation with someone about this subject (36% with family or partner and 33% with friends or colleagues). Thus, PRoTECT can be used both as an examination tool and as a knowledge awareness tool.

Previous studies have examined parents’ preferences for the source of information on managing EH risks. Mothers reported most often receiving information from the Internet, but they preferred their prenatal care providers (26). Approximately 50% of participants in the study indicated they plan to speak with their HCP or already spoke to them about EH risks. Of those who do not plan to consult with their HCP, most reported they had other important issues they wanted to raise, or they did not know what they should ask.

Some HCPs may still be missing certain knowledge or skills pertaining specifically to the threats and uncertainties posed by toxic chemicals on their patients’ health (27). Medical schools and post-graduate programs often provide limited training in EH for physicians (28), which may contribute to limited awareness about the scope of the problem or how to help their patients mitigate risks (28). However, recent efforts have been made to address this issue, such as integrating EH into post-secondary education and physician training as well as establishing educational resources within clinics (29, 30).

This study used CloudResearch’s Prime Panels® to recruit participants for our Internet-based study. This convenience sample may be less representative than a convenience sample recruited through traditional means. In our case, participants were required to have access to the Internet, and this accessibility may have provided them with greater exposure to information about children’s EH. However, because Prime Panels® was used for this study, we were able to recruit a large, international pool of participants across five countries. This allowed us to examine patterns and preferences about the impact of toxic chemicals on neurodevelopment, concerns about exposure, and barriers to making change at a global level. There is also potential sampling bias due to the higher proportion of participants with higher education and socioeconomic status, which may impact perspectives on chemical exposure risks. However, despite this, supplemental analyses, which included stratification by various demographic factors (i.e., gender, age, SES, race) did not yield large differences in attitudes. Additionally, more U.S. residents from the experimental group participated in the 6-week follow-up than the control group. This may have impacted the interpretation of data regarding perceptions of governmental involvement, given the differing governmental representations. Also, the retention rate over the 6-week follow up was approximately 31.1%, indicating a significant drop. While this retention rate is in line with other data collection survey cites (15), it raises the possibility of selection bias. Lastly, some participants were excluded if they failed the attention check during the survey or left midway through the video. This exclusion may have inadvertently removed less engaged individuals, resulting in a sample that was more attentive and potentially overestimating the overall effect of the video on a general population. However, of the 7,070 participants who answered the attention check question, approximately 96% passed, showing an overall high level of understandability by a majority of participants. Additionally, the exclusion of participants who failed the attention checks throughout the survey or left midway through the video may have impacted the randomization. However, this exclusion criteria was applied uniformly across both groups, and the large sample size likely minimized any substantial imbalance.

## 5 Conclusion

This study attempted to illuminate environmental chemicals as risk factors for neurodevelopmental disorders in parents and people of parenting age, with the ultimate goal of finding ways to reduce their prevalence. Substantial resources are spent on interventions for children with NDDs; too little is devoted to prevention – which is what the participants indicated they preferred. One way to prevent new cases of NDDs is to reduce widespread exposure to toxic chemicals. In this study, participants endorsed great interest in learning more about risks posed by toxic chemicals and ways to reduce exposure. Watching a video on toxic chemicals and neurodevelopment was associated with greater knowledge of developmental neurotoxicity and preferences towards prevention and reducing exposures. However, systemic barriers, such as the high cost of nontoxic products and not knowing where or how to purchase nontoxic products, impacted participants’ abilities to make changes. While education is a critical first step in promoting awareness towards reducing exposure, it is not sufficient to protect the next generation of children and prevent another epidemic of poisoning from lead, chemicals in plastics, or other toxic chemicals. Studies, like the current one, may lead to increased awareness which can encourage parents and HCPs to advocate for stricter regulations of toxic chemicals.

## Data Availability

The original contributions presented in the study are included in the article/Supplementary material, further inquiries can be directed to the corresponding author.
