# Corrigendum: Global randomized controlled trial of knowledge translation of children's environmental health

**DOI:** 10.3389/fpubh.2025.1608418

**Published:** 2025-05-14

**Authors:** Rivka Green, Christine Till, Jana El-Sabbagh, Allya DaCosta, Erica Phipps, Carly V. Goodman, David B. Flora, Bruce Lanphear

**Affiliations:** ^1^The Hospital for Sick Children, Toronto, ON, Canada; ^2^Faculty of Health, York University, Toronto, ON, Canada; ^3^Canadian Partnership for Children's Health and Environment (CPCHE), Ottawa, ON, Canada; ^4^Faculty of Health Sciences, Simon Fraser University, Vancouver, BC, Canada

**Keywords:** brain development, developmental neurotoxicology, knowledge translation, prevention, health literacy

In the published article, there was an error in the **Introduction section**. One of the sentences in the introduction for Global Randomized Controlled Trial of Knowledge Translation of Children's Environmental Health currently reads:

“The current study examined **women of childbearing age**'s knowledge of toxic chemicals and their knowledge of how to reduce children's exposure using the Prevention of Toxic Chemicals in the Environment for Children Tool (PRoTECT) questionnaire (13).”

The correct statement is:

“The current study examined **people of childbearing age**'s knowledge of toxic chemicals and their knowledge of how to reduce children's exposure using the Prevention of Toxic Chemicals in the Environment for Children Tool (PRoTECT) questionnaire (13).”

The authors apologize for this error and state that this does not change the scientific conclusions of the article in any way. The original article has been updated.

