# Supplementary material for: Global randomized controlled trial of knowledge translation of children’s environmental health
Source: Front Public Health. 2025 Mar 20;13:1502006. doi: 10.3389/fpubh.2025.1502006 (PMC11965636; doi:10.3389/fpubh.2025.1502006)
Supplement: Supplementary file 1 [file Table_1.docx]

##### **Supplemental Table 1.** *Confirmatory Factor Analysis for PRoTECT*

|  | Question Text | Estimate | Std. Err |
| --- | --- | --- | --- |
| Factor 1*: Preferences to lower exposure and increase prevention efforts* | | | |
| Question 6 | When it comes to addressing developmental conditions affecting children, most governments spend the majority of the health budget on management and treatment of these conditions. I think governments should spend more of their budget to find ways to prevent children from developing these conditions. | 0.514 | 0.008 |
| Question 9 | I want to learn more about how to reduce children’s exposure to toxic chemicals. | 0.610 | 0.008 |
| Question 10 | Of all the sources of information about health impacts from toxic chemicals, I trust information coming from scientists who study them. | 0.448 | 0.008 |
| Question 11 | Exposure to toxic chemicals is particularly harmful to babies and children. | 0.538 | 0.008 |
| Question 13 | More children would benefit by regulating and reducing toxic chemicals to **prevent** developmental conditions than the number of children who benefit from **treatment** of these conditions. | 0.511 | 0.007 |
| Question 15 | My government should strengthen their policies and programs to make sure that consumer products do not contain toxic chemicals that are harmful to children. | 0.564 | 0.007 |
| Question 16 | If I knew how to reduce children’s exposure to toxic chemicals, I would try to do it. | 0.581 | 0.008 |
| Question 17 | I try to purchase products that do not contain toxic chemicals that may be harmful to my family. | 0.543 | 0.008 |
| Factor 2: *Knowledge of regulations of toxic chemicals by government and industry* | | | |
| Question 2 | Most governments spend about the same amount to **prevent** developmental conditions as they spend to **treat** these conditions. | 0.862 | 0.010 |
| Question 3 | All parents have equal opportunities to protect their children from toxic chemicals like pesticides or heavy metals, regardless of income level, race and ethnicity, or where they live. | 0.990 | 0.011 |
| Question 4 | My government has effective regulations to ensure that food and personal care products do not contain harmful levels of toxic chemicals. | 0.679 | 0.010 |
| Question 7 | If toxic chemicals were a threat to my family’s health, my pediatrician, doctor, or health care provider would have told me about it. | 0.692 | 0.010 |
| Question 12 | I trust that most companies make products that don’t contain harmful levels of toxic chemicals. | 0.768 | 0.011 |
| Factor 3: *Knowledge of developmental neurotoxicity* | | | |
| Question 1 | Toxic chemicals in our day-to-day lives, like air pollution or lead in drinking water, can increase a child’s risk of developing conditions like ADHD or autism. | 0.737 | 0.012 |
| Question 5: | Reducing exposure to toxic chemicals during pregnancy and in early childhood can help lower a child’s risk of developing a condition like ADHD or autism. | 0.751 | 0.010 |
| Question 8 | Exposure to toxic chemicals during pregnancy can increase a child’s risk of having a developmental condition. | 0.596 | 0.009 |
| Question 14 | Toxic chemicals can be detected in the blood of most pregnant women. | 0.387 | 0.008 |
