# Supplementary material for: Global randomized controlled trial of knowledge translation of children’s environmental health
Source: Front Public Health. 2025 Mar 20;13:1502006. doi: 10.3389/fpubh.2025.1502006 (PMC11965636; doi:10.3389/fpubh.2025.1502006)
Supplement: Supplementary file 2 [file Table_2.docx]

##### **Supplemental Table 2.** *Mean(SD) and Percentage for Responses on Each Question of PRoTECT*

| Question | Question Text | M(SD) | Strongly disagree  (1) | Disagree | Neither agree nor disagree | Agree | Strongly agree  (5) |
| --- | --- | --- | --- | --- | --- | --- | --- |
| Theme one:  *Preferences to lower exposure and increase prevention* | | 4.21(0.59) |  |  |  |  |  |
| Question 6 | When it comes to addressing developmental conditions affecting children, most governments spend the majority of the health budget on management and treatment of these conditions. I think governments should spend more of their budget to find ways to prevent children from developing these conditions. | 4.02(0.99) | 2.40 | 4.76 | 19.33 | 35.87 | 37.64 |
| Question 9 | I want to learn more about how to reduce children’s exposure to toxic chemicals. | 4.06(0.99) | 2.33 | 4.64 | 18.74 | 33.41 | 40.88 |
| Question 10 | Of all the sources of information about health impacts from toxic chemicals, I trust information coming from scientists who study them. | 4.20(0.88) | 1.08 | 3.07 | 14.99 | 36.52 | 44.34 |
| Question 11 | Exposure to toxic chemicals is particularly harmful to babies and children. | 4.41(0.83) | 0.72 | 2.28 | 10.90 | 27.46 | 58.64 |
| Question 13 | More children would benefit by regulating and reducing toxic chemicals to **prevent**  developmental conditions than the number of children who benefit from **treatment** of these conditions. | 4.10(0.88) | 0.98 | 2.50 | 20.63 | 37.28 | 38.61 |
| Question 15 | My government should strengthen their policies and programs to make sure that consumer products do not contain toxic chemicals that are harmful to children. | 4.35(0.83) | 0.93 | 1.65 | 12.90 | 30.61 | 53.91 |
| Question 16 | If I knew how to reduce children’s exposure to toxic chemicals, I would try to do it. | 4.39(0.84) | 0.81 | 2.17 | 12.15 | 26.82 | 58.05 |
| Question 17 | I try to purchase products that do not contain toxic chemicals that may be harmful to my family. | 4.17(0.92) | 1.15 | 3.66 | 17.54 | 32.47 | 45.18 |

| Question | Question Text | M(SD) | Strongly agree  (1) | Agree | Neither agree nor disagree | Disagree | Strongly disagree (5) |  |  |  |
| --- | --- | --- | --- | --- | --- | --- | --- | --- | --- | --- |
| Theme two:  *Attitudes towards regulations of toxic chemicals by government and industry* | | 2.83(0.91) |  |  |  |  |  |  |  |  |
| Question 2 | Most governments spend about the same amount to **prevent** developmental conditions as they spend to **treat** these conditions. | 3.05(1.23) | 11.9 | 23.68 | 25.52 | 25.08 | 13.83 |  |  |  |
| Question 3 | All parents have equal opportunities to protect their children from toxic chemicals like pesticides or heavy metals, regardless of income level, race and ethnicity, or where they live. | 2.99(1.51) | 23.94 | 19.37 | 13.46 | 20.39 | 22.83 |  |  |  |
| Question 4 | My government has effective regulations to ensure that food and personal care products do not contain harmful levels of toxic chemicals. | 2.63(1.15) | 15.63 | 36.69 | 24.14 | 16.08 | 7.46 |  |  |  |
| Question 7 | If toxic chemicals were a threat to my family’s health, my pediatrician, doctor, or health care provider would have told me about it. | 2.60(1.17) | 20.10 | 30.06 | 25.80 | 17.86 | 6.17 |  |  |  |
| Question 12 | I trust that most companies make products that don’t contain harmful levels of toxic chemicals. | 2.87(1.24) | 14.63 | 28.30 | 23.42 | 22.28 | 11.37 |  |  |  |
| Question | Question Text | M(SD) | Strongly  disagree  (1) | Disagree | Neither Agree Strongly  agree nor agree  disagree (5) | | | | Agree | Strongly agree  (5) |
| Theme three:  *Knowledge of developmental neurotoxicity* | | 3.90(0.72) |  |  |  |  |  |  |  |  |
| Question 1 | Toxic chemicals in our day-to-day lives, like air pollution or lead in drinking water, can increase a child’s risk of developing conditions like ADHD or autism. | 3.83(1.14) | 5.19 | 7.68 | 20.49 | 32.04 | 34.61 |  |  |  |
| Question 5 | Reducing exposure to toxic chemicals during pregnancy and in early childhood can help lower a child’s risk of developing a condition like ADHD or autism. | 3.96(1.00) | 2.69 | 4.90 | 21.85 | 35.06 | 35.50 |  |  |  |
| Question 8 | Exposure to toxic chemicals during pregnancy can increase a child’s risk of having a developmental condition. | 4.25(0.86) | 0.95 | 2.42 | 14.89 | 34.64 | 47.10 |  |  |  |
| Question 14 | Toxic chemicals can be detected in the blood of most pregnant women. | 3.57(0.89) | 1.31 | 6.76 | 42.58 | 32.57 | 16.78 |  |  |  |
