# Supplementary material for: Global randomized controlled trial of knowledge translation of children’s environmental health
Source: Front Public Health. 2025 Mar 20;13:1502006. doi: 10.3389/fpubh.2025.1502006 (PMC11965636; doi:10.3389/fpubh.2025.1502006)
Supplement: Supplementary file 3 [file Table_3.docx]

##### **Supplemental Table 3.** *Responses on PRoTECT Themes by Demographic Characteristics*

|  | M(SD) | | |
| --- | --- | --- | --- |
| Demographic Factor | Theme one: *Preferences to lower exposure and increase prevention* | Theme two:  *Attitudes Towards Regulations of Toxic Chemicals* | Theme three: *Knowledge of Developmental Neurotoxicity* |
| Gender |  |  |  |
| Female | 4.21 (0.58) | 2.92 (0.91) | 3.82 (0.74) |
| Male | 4.21 (0.60) | 2.72 (0.90) | 4.00 (0.67) |
| Non-Binary | 4.07 (0.66) | 3.32 (0.91) | 3.47 (0.86) |
| Country |  |  |  |
| Canada | 4.13 (0.61) | 2.88 (0.87) | 3.81 (0.71) |
| Australia | 4.12 (0.60) | 2.70 (0.79) | 3.72 (0.72) |
| India | 4.49 (0.51) | 2.30 (0.87) | 4.35 (0.55) |
| United Kingdom | 4.03 (0.58) | 2.81 (0.79) | 3.61 (0.70) |
| United States | 4.24 (0.57) | 3.26 (0.88) | 3.93 (0.68) |
| Ethnic Groups |  |  |  |
| White | 4.12 (0.58) | 3.02 (0.86) | 3.72 (0.72) |
| Black | 4.25 (0.58) | 2.95 (0.93) | 4.02 (0.67) |
| South Asian | 4.47 (0.5) | 2.37 (0.88) | 4.32 (0.56) |
| Other | 4.18 (0.63) | 2.76 (0.90) | 3.92 (0.7) |
| Age (Years) |  |  |  |
| 18-24 years | 4.14 (0.63) | 2.68 (0.85) | 3.85 (0.75) |
| 25-32 years | 4.24 (0.57) | 2.81 (0.92) | 3.92 (0.72) |
| 35-39 years | 4.23 (0.59) | 2.92 (0.92) | 3.92 (0.69) |
| 40+ years | 4.19 (0.59) | 2.87 (0.92) | 3.88 (0.71) |
| Level of Education |  |  |  |
| High school or less | 4.06 (0.63) | 2.75 (0.83) | 3.76 (0.73) |
| Some college or university, no degree/diploma | 4.16 (0.58) | 3.00 (0.87) | 3.82 (0.73) |
| Bachelor's degree or diploma | 4.26 (0.56) | 2.90 (0.91) | 3.93 (0.69) |
| Master's, doctorate or professional degree | 4.36 (0.57) | 2.65 (1.00) | 4.11 (0.68) |
| Children |  |  |  |
| Yes, I have children | 4.27 (0.57) | 2.74 (0.93) | 3.93 (0.72) |
| No, I don’t have children | 4.17 (0.6) | 2.90 (0.89) | 3.88 (0.72) |
| Pregnancy Status (No (%)) |  |  |  |
| Pregnant | 4.25 (0.6) | 2.36 (0.91) | 4.07 (0.68) |
| Non-pregnant | 4.28 (0.57) | 2.79 (0.92) | 3.91 (0.72) |
| Identified Developmental Conditions (Participants’ Children) |  |  |  |
| No | 4.29 (0.57) | 2.68 (0.92) | 3.98 (0.69) |
| Yes | 4.21 (0.58) | 2.94 (0.93) | 3.75 (0.80) |
