# Supplementary material for: Global randomized controlled trial of knowledge translation of children’s environmental health
Source: Front Public Health. 2025 Mar 20;13:1502006. doi: 10.3389/fpubh.2025.1502006 (PMC11965636; doi:10.3389/fpubh.2025.1502006)
Supplement: Supplementary file 4 [file Table_4.docx]

**Supplemental Table 4.** *Demographic Characteristics of Participants at Six-Week Follow-Up by Group Status*

|  | Six-week follow-up | |
| --- | --- | --- |
|  | Control Group | Experimental Group |
| *n* | 2206 | 2636 |
| Gender (frequency (%)) |  |  |
| Female | 1097 (49.73) | 1364 (51.75) |
| Male | 1092 (49.50) | 1246 (47.27) |
| Non-Binary | 12 (0.54) | 20 (0.76) |
| Country (frequency (%)) |  |  |
| Canada | 187 (8.48) | 198 (7.51) |
| Australia | 250 (11.33) | 227 (8.61) |
| India | 541 (24.52) | 474 (17.98) |
| United Kingdom | 277 (12.56) | 252 (9.56) |
| United States | 951 (43.11) | 1485 (56.34) |
| Ethnicity (frequency (%)) |  |  |
| White | 1118 (50.68) | 1361 (51.63) |
| Black | 187 (8.48) | 253 (9.60) |
| South Asian | 478 (21.67) | 473 (17.94) |
| Other | 379 (17.18) | 510 (19.35) |
| Location (frequency (%)) |  |  |
| Major City | 781 (35.40) | 885 (33.57) |
| Suburban Edges | 714 (32.37) | 829 (31.45) |
| Major Town | 242 (10.97) | 305 (11.57) |
| Small Town | 399 (18.09) | 524 (19.88) |
| Remote | 53 (2.40) | 73 (2.77) |
| Age in years (M, SD) | 33.48 (8.16) | 33.36 (7.47) |
| Level of Education (frequency (%)) |  |  |
| High school or less | 369 (16.73) | 388 (14.72) |
| Some college or university, no  degree/diploma | 344 (15.59) | 453 (17.19) |
| Bachelor’s degree or diploma | 1035 (46.92) | 1270 (48.18) |
| Master’s, doctorate, or professional  degree | 440 (19.95) | 516 (19.58) |
| Employment (frequency (%)) |  |  |
| Full-time | 1223 (55.44) | 1498 (56.83) |
| Part-time | 288 (13.06) | 309 (11.72) |
| Other (i.e., retired, student, self-  employed, unemployed) | 680 (30.83) | 810 (30.73) |
| Politics |  |  |
| Mean (SD), Median | 4.93 (2.64), 5 | 4.58 (2.65), 5 |
| *Parental Questions* |  |  |
| Marital Status (frequency (%)) |  |  |
| Single | 1103 (50) | 1351 (51.25) |
| Married / Common Law | 1007 (46.65) | 1146 (43.47) |
| Separated / Divorced or Widowed | 96 (4.25) | 139 (5.27) |
| Children (frequency (%)) |  |  |
| Yes, I have children | 1014 (45.97) | 1149 (43.59) |
| No, I don’t have children | 1192 (54.03) | 1487 (56.41) |
| Pregnancy Status (frequency (%)) |  |  |
| Pregnant | 122 (12.12) | 97 (8.46) |
| Non-pregnant | 885 (87.88) | 1049 (91.54) |
| Endorsed Developmental Conditions (Participants’ Children) (frequency (%)) |  |  |
| No | 819 (81.57) | 910 (80.32) |
| Yes | 152 (15.14) | 193 (17.03) |
| I don’t know | 26 (2.59) | 26 (2.29) |
| Other | 7 (0.70) | 4 (0.35) |
| Identified Developmental Conditions (Participants) (frequency (%)) |  |  |
| No | 892 (88.67) | 1009 (88.51) |
| Yes | 94 (9.34) | 115 (10.09) |
| I don’t know | 20 (1.99) | 16 (1.40) |
