# Supplementary material for: Global randomized controlled trial of knowledge translation of children’s environmental health
Source: Front Public Health. 2025 Mar 20;13:1502006. doi: 10.3389/fpubh.2025.1502006 (PMC11965636; doi:10.3389/fpubh.2025.1502006)
Supplement: Supplementary file 5 [file Table_5.docx]

**Supplemental Table 5.** *Higher Responses on PRoTECT Questions Collapsed Across Groups and Stratified by Country, Pregnancy Status, Levels of Education, and Age by Group Status*

| Question | Question Text | | Demographic Variable | | Percent of Correct Responses  (Strongly Agree) | | | | | |
| --- | --- | --- | --- | --- | --- | --- | --- | --- | --- | --- |
|  |  |  |  |  | Control group | | | Experimental group | | |
|  |  |  |  |  | Pre | Post | % Change | Pre | Post | % Change |
| Theme 1: *Preferences to lower exposure and increase prevention efforts* | | | | | | | | | | |
| Question 6 | When it comes to addressing developmental conditions affecting children, most governments spend the majority of the health budget on management and treatment of these conditions. I think governments should spend more of their budget to find ways to prevent children from developing these conditions. | | Collapsed Across Groups | | 37.35 | 41.01 | 3.66 | 37.85 | 45.39 | 7.54* |
|  |  |  | Country |  |  |  |  |  |  |  |
|  |  |  |  | Canada | 13.77 | 6.12 | -7.65  *** | 15.83 | 6.52 | -9.31  *** |
|  |  |  |  | Australia | 12.87 | 7.65 | -5.22 *** | 13.66 | 7.35 | -6.31 *** |
|  |  |  |  | India | 28.89 | 32.46 | 3.57 | 29.74 | 20.81 | -8.93  *** |
|  |  |  |  | United Kingdom | 11.22 | 8.2 | -3.02 | 11.8 | 6.03 | -5.77  *** |
|  |  |  |  | United States | 33.25 | 45.57 | 12.32** | 28.98 | 59.29 | 30.31** |
|  |  |  | Pregnancy Status |  |  |  |  |  |  |  |
|  |  |  |  | Yes | 11.95 | 10.54 | -1.41 | 11.76 | 6.49 | -5.27  *** |
|  |  |  |  | No | 88.05 | 89.46 | 1.41 | 88.24 | 93.51 | 5.27* |
|  |  |  | Levels of Education |  |  |  |  |  |  |  |
|  |  |  |  | High school or less | 20.34 | 14.33 | -6.01  *** | 18.86 | 13.59 | -5.27  *** |
|  |  |  |  | Some college or university, no degree/  diploma | 15.61 | 15.44 | -0.17 | 15.68 | 16.65 | 0.97 |
|  |  |  |  | Bachelor's degree or diploma | 42.27 | 46.53 | 4.26 | 43.02 | 48.38 | 5.36* |
|  |  |  |  | Master's, doctorate or professional degree | 21.77 | 23.7 | 1.93 | 22.45 | 21.38 | -1.07 |
|  |  |  | Age |  |  |  |  |  |  |  |
|  |  |  |  | 18-24 years | 19.4 | 13.66 | -5.74  *** | 21.18 | 12.47 | -8.71  *** |
|  |  |  |  | 25-32 years | 31.37 | 31.69 | 0.32 | 31.93 | 31.63 | -0.3 |
|  |  |  |  | 33-39 years | 28.77 | 32.68 | 3.91 | 28.1 | 33.2 | 5.1* |
|  |  |  |  | 40+ years | 20.46 | 21.97 | 1.51 | 18.78 | 22.71 | 3.93 |
| Question 9 | I want to learn more about how to reduce children’s exposure to toxic chemicals. | | Collapsed Across Groups | | 39.79 | 43.33 | 3.54 | 41.66 | 42.01 | 0.35 |
|  |  |  | Country |  |  |  |  |  |  |  |
|  |  |  |  | Canada | 13.69 | 6.96 | -6.73  *** | 16.66 | 6.46 | -10.2  **** |
|  |  |  |  | Australia | 12.21 | 7.69 | -4.52 | 13.69 | 7.45 | -6.24  *** |
|  |  |  |  | India | 32.98 | 40.33 | 7.35* | 32.74 | 29.62 | -3.12 |
|  |  |  |  | United Kingdom | 12.07 | 9.36 | -2.71 | 11.77 | 6.82 | -4.95 |
|  |  |  |  | United States | 29.05 | 35.65 | 6.6* | 25.15 | 49.64 | 24.49** |
|  |  |  | Pregnancy Status |  |  |  |  |  |  |  |
|  |  |  |  | Yes | 12.49 | 12.7 | 0.21 | 11.68 | 7.43 | -4.25 |
|  |  |  |  | No | 87.51 | 87.3 | -0.21 | 88.32 | 92.57 | 4.25 |
|  |  |  | Levels of Education |  |  |  |  |  |  |  |
|  |  |  |  | High school or less | 20.24 | 13.03 | -7.21  *** | 18.46 | 13.05 | -5.41  *** |
|  |  |  |  | Some college or university, no degree/  diploma | 15.1 | 13.76 | -1.34 | 14.6 | 14.04 | -0.56 |
|  |  |  |  | Bachelor's degree or diploma | 42.15 | 48 | 5.85* | 42.92 | 47.79 | 4.87 |
|  |  |  |  | Master's, doctorate or professional degree | 22.51 | 25.21 | 2.7 | 24.02 | 25.11 | 1.09 |
|  |  |  | Age |  |  |  |  |  |  |  |
|  |  |  |  | 18-24 years | 20.36 | 15.9 | -4.46 | 21.81 | 14.18 | -7.63  *** |
|  |  |  |  | 25-32 years | 34.97 | 31.91 | -3.06 | 33.3 | 33.21 | -0.09 |
|  |  |  |  | 33-39 years | 26.87 | 31.6 | 4.73 | 28.1 | 32.23 | 4.13 |
|  |  |  |  | 40+ years | 17.8 | 20.58 | 2.78 | 16.79 | 20.38 | 3.59 |
| Question 10 | Of all the sources of information about health impacts from toxic chemicals, I trust information coming from scientists who study them. | | Collapsed Across Groups | | 43.69 | 45.74 | 2.05 | 44.82 | 47.06 | 2.24 |
|  |  |  | Country |  |  |  |  |  |  |  |
|  |  |  |  | Canada | 14.51 | 6.21 | -8.3  *** | 16.58 | 6.17 | -10.41  **** |
|  |  |  |  | Australia | 14.3 | 8.97 | -5.33  *** | 14.98 | 7.22 | -7.76  *** |
|  |  |  |  | India | 27.6 | 33.5 | 5.9* | 28.02 | 23.34 | -4.68 |
|  |  |  |  | United Kingdom | 13.33 | 8.87 | -4.46 | 12.71 | 6.5 | -6.21  *** |
|  |  |  |  | United States | 30.26 | 42.46 | 12.2** | 27.71 | 56.78 | 29.07** |
|  |  |  | Pregnancy Status |  |  |  |  |  |  |  |
|  |  |  |  | Yes | 12.89 | 9.95 | -2.94 | 11.63 | 8.38 | -3.25 |
|  |  |  |  | No | 87.11 | 90.05 | 2.94 | 88.37 | 91.62 | 3.25 |
|  |  |  | Levels of Education |  |  |  |  |  |  |  |
|  |  |  |  | High school or less | 19.93 | 13.01 | -6.92  *** | 18.52 | 11.41 | -7.11  *** |
|  |  |  |  | Some college or university, no degree/  diploma | 14.51 | 14.9 | 0.39 | 15 | 16.16 | 1.16 |
|  |  |  |  | Bachelor's degree or diploma | 42.95 | 48.36 | 5.41* | 43.34 | 48.63 | 5.29* |
|  |  |  |  | Master's, doctorate or professional degree | 22.62 | 23.73 | 1.11 | 23.14 | 23.79 | 0.65 |
|  |  |  | Age |  |  |  |  |  |  |  |
|  |  |  |  | 18-24 years | 21.32 | 17.54 | -3.78 | 22.82 | 13.23 | -9.59  *** |
|  |  |  |  | 25-32 years | 32.32 | 29.56 | -2.76 | 33.99 | 33.76 | -0.23 |
|  |  |  |  | 33-39 years | 27.56 | 33.2 | 5.64* | 27.02 | 32.88 | 5.86* |
|  |  |  |  | 40+ years | 18.79 | 19.7 | 0.91 | 16.17 | 20.13 | 3.96 |
| Question 11 | Exposure to toxic chemicals is particularly harmful to babies and children. | | Collapsed Across Groups | | 59.03 | 62.94 | 3.91 | 58.37 | 67.51 | 9.14* |
|  |  |  | Country |  |  |  |  |  |  |  |
|  |  |  |  | Canada | 15.63 | 6.88 | -8.75  *** | 17.71 | 6.49 | -11.22  **** |
|  |  |  |  | Australia | 15.11 | 10.03 | -5.08  *** | 15.5 | 7.67 | -7.83  *** |
|  |  |  |  | India | 22.19 | 26.07 | 3.88 | 22.49 | 17.29 | -5.2  *** |
|  |  |  |  | United Kingdom | 12.98 | 9.81 | -3.17 | 12.97 | 7.11 | -5.86  *** |
|  |  |  |  | United States | 34.09 | 47.21 | 13.12** | 31.32 | 61.44 | 30.12** |
|  |  |  | Pregnancy Status |  |  |  |  |  |  |  |
|  |  |  |  | Yes | 10.37 | 10.46 | 0.09 | 10.33 | 7.67 | -2.66 |
|  |  |  |  | No | 89.63 | 89.54 | -0.09 | 89.67 | 92.33 | 2.66 |
|  |  |  | Levels of Education |  |  |  |  |  |  |  |
|  |  |  |  | High school or less | 22.56 | 14.37 | -8.19  *** | 20.6 | 12.65 | -7.95  *** |
|  |  |  |  | Some college or university, no degree/  diploma | 16.91 | 16.03 | -0.88 | 17.11 | 16.86 | -0.25 |
|  |  |  |  | Bachelor's degree or diploma | 41.63 | 48.23 | 6.6* | 42.72 | 49.52 | 6.8* |
|  |  |  |  | Master's, doctorate or professional degree | 18.9 | 21.37 | 2.47 | 19.57 | 20.97 | 1.4 |
|  |  |  | Age |  |  |  |  |  |  |  |
|  |  |  |  | 18-24 years | 18.76 | 13.11 | -5.65  *** | 20.59 | 11.58 | -9.01  *** |
|  |  |  |  | 25-32 years | 31.74 | 29.87 | -1.87 | 32.63 | 32.01 | -0.62 |
|  |  |  |  | 33-39 years | 28.99 | 33.95 | 4.96 | 27.97 | 33.63 | 5.66* |
|  |  |  |  | 40+ years | 20.51 | 23.07 | 2.56 | 18.81 | 22.78 | 3.97 |
| Question 13 | More children would benefit by regulating and reducing toxic chemicals to **prevent** developmental conditions than the number of children who benefit from **treatment** of these conditions. | | Collapsed Across Groups | | 39.27 | 44.02 | 4.75 | 38.13 | 47.14 | 9.01* |
|  |  |  | Country |  |  |  |  |  |  |  |
|  |  |  |  | Canada | 13.66 | 6.26 | -7.4  *** | 17.15 | 6.9 | -10.25  **** |
|  |  |  |  | Australia | 13.81 | 9.13 | -4.68 | 13.87 | 6.82 | -7.05  *** |
|  |  |  |  | India | 26.3 | 30.15 | 3.85 | 27.32 | 21.51 | -5.81  *** |
|  |  |  |  | United Kingdom | 13.36 | 10.05 | -3.31 | 12.77 | 5.7 | -7.07  *** |
|  |  |  |  | United States | 32.88 | 44.41 | 11.53  ** | 28.89 | 59.07 | 30.18  ** |
|  |  |  | Pregnancy Status |  |  |  |  |  |  |  |
|  |  |  |  | Yes | 11.97 | 11.97 | 0 | 11.54 | 6.77 | -4.77 |
|  |  |  |  | No | 88.03 | 88.03 | 0 | 88.46 | 93.23 | 4.77 |
|  |  |  | Levels of Education |  |  |  |  |  |  |  |
|  |  |  |  | High school or less | 20.92 | 14.99 | -5.93  *** | 19.91 | 12.88 | -7.03  *** |
|  |  |  |  | Some college or university, no degree/  diploma | 15.03 | 15.62 | 0.59 | 14.74 | 16.59 | 1.85 |
|  |  |  |  | Bachelor's degree or diploma | 43.05 | 45.71 | 2.66 | 41.99 | 47.99 | 6* |
|  |  |  |  | Master's, doctorate or professional degree | 20.99 | 23.68 | 2.69 | 23.35 | 22.54 | -0.81 |
|  |  |  | Age |  |  |  |  |  |  |  |
|  |  |  |  | 18-24 years | 18.51 | 13.54 | -4.97 | 19.85 | 12.84 | -7.01  *** |
|  |  |  |  | 25-32 years | 31.6 | 29.85 | -1.75 | 32.37 | 31.14 | -1.23 |
|  |  |  |  | 33-39 years | 28.14 | 33.13 | 4.99 | 28.92 | 32.74 | 3.82 |
|  |  |  |  | 40+ years | 21.75 | 23.49 | 1.74 | 18.87 | 23.27 | 4.4 |
| Question 15 | My government should strengthen their policies and programs to make sure that consumer products do not contain toxic chemicals that are harmful to children. | | Collapsed Across Groups | | 53.32 | 56.52 | 3.20 | 54.34 | 60.58 | 6.24* |
|  |  |  | Country |  |  |  |  |  |  |  |
|  |  |  |  | Canada | 14.37 | 6.97 | -7.4  *** | 16.66 | 7.13 | -9.53  *** |
|  |  |  |  | Australia | 13.95 | 10.01 | -3.94 | 14.68 | 8.19 | -6.49  *** |
|  |  |  |  | India | 24.37 | 29.86 | 5.49* | 25.48 | 20.44 | -5.04  *** |
|  |  |  |  | United Kingdom | 14.28 | 9.93 | -4.35 | 14.23 | 6.56 | -7.67  *** |
|  |  |  |  | United States | 33.04 | 43.23 | 10.19** | 28.95 | 57.69 | 28.74** |
|  |  |  | Pregnancy Status |  |  |  |  |  |  |  |
|  |  |  |  | Yes | 11.3 | 10.39 | -0.91 | 10 | 6.4 | -3.6 |
|  |  |  |  | No | 88.7 | 89.61 | 0.91 | 90 | 93.6 | 3.6 |
|  |  |  | Levels of Education |  |  |  |  |  |  |  |
|  |  |  |  | High school or less | 22.42 | 15.5 | -6.92  *** | 20.55 | 14.06 | -6.49  *** |
|  |  |  |  | Some college or university, no degree/  diploma | 16.73 | 15.74 | -0.99 | 16.17 | 16.89 | 0.72 |
|  |  |  |  | Bachelor's degree or diploma | 41.83 | 47.3 | 5.47* | 42.42 | 48.15 | 5.73* |
|  |  |  |  | Master's, doctorate or professional degree | 19.02 | 21.47 | 2.45 | 20.87 | 20.9 | 0.03 |
|  |  |  | Age |  |  |  |  |  |  |  |
|  |  |  |  | 18-24 years | 18.7 | 14.41 | -4.29 | 21.24 | 12.94 | -8.3  *** |
|  |  |  |  | 25-32 years | 30.95 | 29.7 | -1.25 | 31.85 | 30.81 | -1.04 |
|  |  |  |  | 33-39 years | 29.09 | 33.23 | 4.14 | 27.71 | 33.06 | 5.35* |
|  |  |  |  | 40+ years | 21.26 | 22.66 | 1.4 | 19.2 | 23.19 | 3.99 |
| Question 16 | If I knew how to reduce children’s exposure to toxic chemicals, I would try to do it. | | Collapsed Across Groups | | 58.18 | 59.89 | 1.71 | 57.95 | 62.69 | 4.74 |
|  |  |  | Country |  |  |  |  |  |  |  |
|  |  |  |  | Canada | 14.9 | 6.8 | -8.1  *** | 16.75 | 6.53 | -10.22  **** |
|  |  |  |  | Australia | 14.8 | 9.75 | -5.05  *** | 15.4 | 7.85 | -7.55  *** |
|  |  |  |  | India | 23.05 | 27.82 | 4.77 | 24.34 | 20 | -4.34 |
|  |  |  |  | United Kingdom | 14.31 | 10.2 | -4.11 | 13.86 | 6.95 | -6.91  *** |
|  |  |  |  | United States | 32.93 | 45.43 | 12.5** | 29.65 | 58.67 | 29.02** |
|  |  |  | Pregnancy Status |  |  |  |  |  |  |  |
|  |  |  |  | Yes | 10.19 | 10.57 | 0.38 | 9.76 | 7.65 | -2.11 |
|  |  |  |  | No | 89.81 | 89.43 | -0.38 | 90.24 | 92.35 | 2.11 |
|  |  |  | Levels of Education |  |  |  |  |  |  |  |
|  |  |  |  | High school or less | 22.25 | 14.84 | -7.41  *** | 20.47 | 13.05 | -7.42  *** |
|  |  |  |  | Some college or university, no degree/  diploma | 16.22 | 16.44 | 0.22 | 16.62 | 16.87 | 0.25 |
|  |  |  |  | Bachelor's degree or diploma | 42.04 | 47.87 | 5.83* | 42.46 | 49.15 | 6.69* |
|  |  |  |  | Master's, doctorate or professional degree | 19.48 | 20.85 | 1.37 | 20.45 | 20.93 | 0.48 |
|  |  |  | Age |  |  |  |  |  |  |  |
|  |  |  |  | 18-24 years | 17.86 | 13.23 | -4.63 | 20.03 | 11.9 | -8.13  *** |
|  |  |  |  | 25-32 years | 31.84 | 30.91 | -0.93 | 32.48 | 32.21 | -0.27 |
|  |  |  |  | 33-39 years | 28.83 | 33.26 | 4.43 | 28.71 | 33.11 | 4.4 |
|  |  |  |  | 40+ years | 21.47 | 22.6 | 1.13 | 18.79 | 22.78 | 3.99 |
| Question 17 | I try to purchase products that do not contain toxic chemicals that may be harmful to my family. | | Collapsed Across Groups | | 45.29 | 44.42 | -0.87 | 45.09 | 46.74 | 1.65 |
|  |  |  | Country |  |  |  |  |  |  |  |
|  |  |  |  | Canada | 14.33 | 7.35 | -6.98  *** | 16.75 | 7.31 | -9.44  *** |
|  |  |  |  | Australia | 14.99 | 9.18 | -5.81  *** | 15.58 | 8.52 | -7.06  *** |
|  |  |  |  | India | 27.03 | 33.27 | 6.24* | 28.62 | 22.32 | -6.3  *** |
|  |  |  |  | United Kingdom | 13.12 | 8.98 | -4.14 | 12.09 | 5.68 | -6.41  *** |
|  |  |  |  | United States | 30.53 | 41.22 | 10.69** | 26.96 | 56.17 | 29.21** |
|  |  |  | Pregnancy Status |  |  |  |  |  |  |  |
|  |  |  |  | Yes | 11.4 | 11.39 | -0.01 | 11.12 | 7.93 | -3.19 |
|  |  |  |  | No | 88.6 | 88.61 | 0.01 | 88.88 | 92.07 | 3.19 |
|  |  |  | Levels of Education |  |  |  |  |  |  |  |
|  |  |  |  | High school or less | 22.03 | 14.59 | -7.44  *** | 19.98 | 13.46 | -6.52  *** |
|  |  |  |  | Some college or university, no degree/  diploma | 15.62 | 15.93 | 0.31 | 15.48 | 14.76 | -0.72 |
|  |  |  |  | Bachelor's degree or diploma | 43.33 | 46.66 | 3.33 | 42.12 | 48.69 | 6.57* |
|  |  |  |  | Master's, doctorate or professional degree | 19.02 | 22.82 | 3.8 | 22.43 | 23.08 | 0.65 |
|  |  |  | Age |  |  |  |  |  |  |  |
|  |  |  |  | 18-24 years | 18.85 | 14.9 | -3.95 | 21.39 | 11.36 | -10.03  **** |
|  |  |  |  | 25-32 years | 32.2 | 30.2 | -2 | 31.79 | 33.04 | 1.25 |
|  |  |  |  | 33-39 years | 28.66 | 33.37 | 4.71 | 27.93 | 33.44 | 5.51* |
|  |  |  |  | 40+ years | 20.29 | 21.53 | 1.24 | 18.89 | 22.16 | 3.27 |
| Question | | Question Text | Demographic Variable | | Percent of Correct Responses  (Disagree and Strongly Disagree combined) | | | | | |
|  |  |  |  |  | Control group | | | Experimental group | | |
|  |  |  |  |  | Pre | Post | % Change | Pre | Post | % Change |
| Theme 2: *Attitudes towards regulations of toxic chemicals by government and industry* | | | | | | | | | | |
| Question 2 | Most governments spend about the same amount to **prevent** developmental conditions as they spend to **treat** these conditions. | | Collapsed Across Groups | | 38.36 | 46.96 | 8.60* | 39.32 | 53.96 | 14.64** |
|  |  |  | Country |  |  |  |  |  |  |  |
|  |  |  |  | Canada | 16.55 | 5.86 | -10.69  **** | 16.85 | 4.94 | -11.91  **** |
|  |  |  |  | Australia | 14.68 | 10.96 | -3.72 | 14.96 | 6.38 | -8.58  *** |
|  |  |  |  | India | 9.46 | 11.34 | 1.88 | 10.99 | 9.06 | -1.93 |
|  |  |  |  | United Kingdom | 17.09 | 12 | -5.09  *** | 15.9 | 7.48 | -8.42  *** |
|  |  |  |  | United States | 42.22 | 59.83 | 17.61** | 41.31 | 72.13 | 30.82** |
|  |  |  | Pregnancy Status |  |  |  |  |  |  |  |
|  |  |  |  | Yes | 7.16 | 6.44 | -0.72 | 7.11 | 6.77 | -0.34 |
|  |  |  |  | No | 92.84 | 55.15 | -37.69  **** | 92.89 | 93.23 | 0.34 |
|  |  |  | Levels of Education |  |  |  |  |  |  |  |
|  |  |  |  | High school or less | 19.83 | 14.58 | -5.25  *** | 18.96 | 12.04 | -6.92  *** |
|  |  |  |  | Some college or university, no degree/  diploma | 18.76 | 16.76 | -2 | 19.53 | 20.23 | 0.7 |
|  |  |  |  | Bachelor's degree or diploma | 45.67 | 49.34 | 3.67 | 44.99 | 49.14 | 4.15 |
|  |  |  |  | Master's, doctorate or professional degree | 15.74 | 19.32 | 3.58 | 16.52 | 18.58 | 2.06 |
|  |  |  | Age |  |  |  |  |  |  |  |
|  |  |  |  | 18-24 years | 15.56 | 10.49 | -5.07  *** | 17.76 | 11.12 | -6.64  *** |
|  |  |  |  | 25-32 years | 30.59 | 29.58 | -1.01 | 31.4 | 31.3 | -0.1 |
|  |  |  |  | 33-39 years | 30.82 | 36.39 | 5.57* | 31.16 | 35.07 | 3.91 |
|  |  |  |  | 40+ years | 23.04 | 23.53 | 0.49 | 19.68 | 22.51 | 2.83 |
| Question 3 | All parents have equal opportunities to protect their children from toxic chemicals like pesticides or heavy metals, regardless of income level, race and ethnicity, or where they live. | | Collapsed Across Groups | | 42.60 | 49.80 | 7.20* | 43.69 | 56.43 | 12.74** |
|  |  |  | Country |  |  |  |  |  |  |  |
|  |  |  |  | Canada | 17.16 | 5.99 | -11.17  **** | 18.93 | 5.92 | -13.01  **** |
|  |  |  |  | Australia | 14.68 | 9.92 | -4.76 | 14.53 | 5.86 | -8.67  *** |
|  |  |  |  | India | 9.01 | 11.8 | 2.79 | 10.58 | 9.61 | -0.97 |
|  |  |  |  | United Kingdom | 17.19 | 11.89 | -5.3  *** | 15.94 | 7.24 | -8.7  *** |
|  |  |  |  | United States | 41.95 | 60.41 | 18.46** | 40.02 | 71.36 | 31.34** |
|  |  |  | Pregnancy Status |  |  |  |  |  |  |  |
|  |  |  |  | Yes | 6.12 | 6.26 | 0.14 | 5.75 | 5.84 | 0.09 |
|  |  |  |  | No | 93.88 | 53.65 | -40.23  **** | 94.25 | 94.16 | -0.09 |
|  |  |  | Levels of Education |  |  |  |  |  |  |  |
|  |  |  |  | High school or less | 20.13 | 14.59 | -5.54  *** | 19.33 | 13.2 | -6.13  *** |
|  |  |  |  | Some college or university, no degree/  diploma | 19.13 | 17.19 | -1.94 | 19.43 | 19.47 | 0.04 |
|  |  |  |  | Bachelor's degree or diploma | 45.17 | 48.52 | 3.35 | 44.92 | 48.91 | 3.99 |
|  |  |  |  | Master's, doctorate or professional degree | 15.57 | 19.7 | 4.13 | 16.32 | 18.42 | 2.1 |
|  |  |  | Age |  |  |  |  |  |  |  |
|  |  |  |  | 18-24 years | 17.64 | 11.89 | -5.75  *** | 19.1 | 12.24 | -6.86  *** |
|  |  |  |  | 25-32 years | 30.67 | 29.13 | -1.54 | 31.58 | 31.67 | 0.09 |
|  |  |  |  | 33-39 years | 29.54 | 36.55 | 7.01* | 30.22 | 34.23 | 4.01 |
|  |  |  |  | 40+ years | 22.15 | 22.43 | 0.28 | 19.1 | 21.86 | 2.76 |
| Question 4 | My government has effective regulations to ensure that food and personal care products do not contain harmful levels of toxic chemicals. | | Collapsed Across Groups | | 23.83 | 28.20 | 4.37 | 23.33 | 34.16 | 10.83** |
|  |  |  | Country |  |  |  |  |  |  |  |
|  |  |  |  | Canada | 14.03 | 4.11 | -9.92  *** | 15.5 | 5.12 | -10.38  **** |
|  |  |  |  | Australia | 7.51 | 5.85 | -1.66 | 9.98 | 4.36 | -5.62  *** |
|  |  |  |  | India | 17.54 | 19.43 | 1.89 | 20.88 | 17.1 | -3.78 |
|  |  |  |  | United Kingdom | 12.68 | 8.69 | -3.99 | 12.76 | 3.81 | -8.95  *** |
|  |  |  |  | United States | 48.25 | 61.93 | 13.68** | 40.88 | 69.61 | 28.73** |
|  |  |  | Pregnancy Status |  |  |  |  |  |  |  |
|  |  |  |  | Yes | 9.42 | 10.91 | 1.49 | 8.21 | 7.63 | -0.58 |
|  |  |  |  | No | 90.58 | 93.45 | 2.87 | 91.79 | 92.37 | 0.58 |
|  |  |  | Levels of Education |  |  |  |  |  |  |  |
|  |  |  |  | High school or less | 19.54 | 14.72 | -4.82 | 18.36 | 11.61 | -6.75  *** |
|  |  |  |  | Some college or university, no degree/  diploma | 18.92 | 13.61 | -5.31  *** | 17.81 | 17.96 | 0.15 |
|  |  |  |  | Bachelor's degree or diploma | 43.8 | 48.73 | 4.93 | 45.11 | 49.95 | 4.84 |
|  |  |  |  | Master's, doctorate or professional degree | 17.74 | 22.94 | 5.2* | 18.72 | 20.48 | 1.76 |
|  |  |  | Age |  |  |  |  |  |  |  |
|  |  |  |  | 18-24 years | 18.95 | 12.8 | -6.15  *** | 20.37 | 13.83 | -6.54  *** |
|  |  |  |  | 25-32 years | 32.68 | 29.7 | -2.98 | 33.14 | 31.7 | -1.44 |
|  |  |  |  | 33-39 years | 27.75 | 36.02 | 8.27* | 27.85 | 32.57 | 4.72 |
|  |  |  |  | 40+ years | 20.62 | 21.48 | 0.86 | 18.64 | 21.9 | 3.26 |
| Question 7 | If toxic chemicals were a threat to my family’s health, my pediatrician, doctor, or health care provider would have told me about it. | | Collapsed Across Groups | | 24.17 | 25.12 | 0.95 | 23.93 | 28.87 | 4.94 |
|  |  |  | Country |  |  |  |  |  |  |  |
|  |  |  |  | Canada | 18.56 | 8.23 | -10.33  **** | 19.13 | 6.78 | -12.35  **** |
|  |  |  |  | Australia | 13.27 | 11.27 | -2 | 14.85 | 6.52 | -8.33  *** |
|  |  |  |  | India | 6.09 | 7.69 | 1. 6 | 7.4 | 6.13 | -1.27 |
|  |  |  |  | United Kingdom | 21.73 | 15.56 | -6.17  *** | 19.48 | 9.52 | -9.96  *** |
|  |  |  |  | United States | 40.35 | 57.25 | 16.9** | 39.14 | 71.06 | 31.92** |
|  |  |  | Pregnancy Status |  |  |  |  |  |  |  |
|  |  |  |  | Yes | 8.8 | 11.45 | 2.65 | 6.72 | 6.37 | -0.35 |
|  |  |  |  | No | 91.2 | 98.09 | 6.89* | 93.28 | 93.63 | 0.35 |
|  |  |  | Levels of Education |  |  |  |  |  |  |  |
|  |  |  |  | High school or less | 23.24 | 17.06 | -6.18  *** | 20.93 | 14.1 | -6.83  *** |
|  |  |  |  | Some college or university, no degree/  diploma | 20.92 | 16.16 | -4.76 | 20.89 | 18.15 | -2.74 |
|  |  |  |  | Bachelor's degree or diploma | 43.12 | 52.42 | 9.3* | 43.22 | 53.39 | 10.17** |
|  |  |  |  | Master's, doctorate or professional degree | 12.72 | 14.36 | 1.64 | 14.96 | 14.36 | -0.6 |
|  |  |  | Age |  |  |  |  |  |  |  |
|  |  |  |  | 18-24 years | 15.28 | 9.3 | -5.98  *** | 15.92 | 10.17 | -5.75  *** |
|  |  |  |  | 25-32 years | 30.19 | 29.52 | -0.67 | 30 | 33.64 | 3.64 |
|  |  |  |  | 33-39 years | 30.55 | 37.57 | 7.02* | 30.81 | 31.03 | 0.22 |
|  |  |  |  | 40+ years | 23.98 | 23.61 | -0.37 | 23.27 | 25.16 | 1.89 |
| Question 12 | I trust that most companies make products that don’t contain harmful levels of toxic chemicals. | | Collapsed Across Groups | | 32.93 | 37.89 | 4.96 | 34.17 | 45.78 | 11.61** |
|  |  |  | Country |  |  |  |  |  |  |  |
|  |  |  |  | Canada | 17.67 | 5.48 | -12.19  **** | 18.03 | 7.02 | -11.01  **** |
|  |  |  |  | Australia | 12.91 | 7.74 | -5.17  *** | 13.27 | 6.11 | -7.16  *** |
|  |  |  |  | India | 13.99 | 17.5 | 3.51 | 16.78 | 14.04 | -2.74 |
|  |  |  |  | United Kingdom | 11.88 | 9.64 | -2.24 | 13.15 | 6.19 | -6.96  *** |
|  |  |  |  | United States | 43.54 | 59.64 | 16.1** | 38.76 | 66.64 | 27.88** |
|  |  |  | Pregnancy Status |  |  |  |  |  |  |  |
|  |  |  |  | Yes | 8.56 | 8.13 | -0.43 | 8.35 | 7.71 | -0.64 |
|  |  |  |  | No | 91.44 | 69.65 | -21.79  *** | 91.65 | 92.29 | 0.64 |
|  |  |  | Levels of Education |  |  |  |  |  |  |  |
|  |  |  |  | High school or less | 20.59 | 13.96 | -6.63  *** | 19.02 | 12.33 | -6.69  *** |
|  |  |  |  | Some college or university, no degree/  diploma | 18.65 | 16.23 | -2.42 | 18.11 | 18.71 | 0.6 |
|  |  |  |  | Bachelor's degree or diploma | 44.1 | 49.52 | 5.42* | 45.04 | 49.42 | 4.38 |
|  |  |  |  | Master's, doctorate or professional degree | 16.67 | 20.29 | 3.62 | 17.83 | 19.54 | 1.71 |
|  |  |  | Age |  |  |  |  |  |  |  |
|  |  |  |  | 18-24 years | 17.98 | 10.36 | -7.62  *** | 19.6 | 12.55 | -7.05  *** |
|  |  |  |  | 25-32 years | 30.76 | 31.43 | 0.67 | 31.81 | 32.2 | 0.39 |
|  |  |  |  | 33-39 years | 29.51 | 35.12 | 5.61* | 28.96 | 33.2 | 4.24 |
|  |  |  |  | 40+ years | 21.75 | 23.1 | 1.35 | 19.63 | 22.05 | 2.42 |
| Question | | Question Text | Demographic Variable | | Percent of Correct Responses  (Strongly Agree) | | | | | |
|  |  |  |  |  | Control group | | | Experimental group | | |
|  |  |  |  |  | Pre | Post | % Change | Pre | Post | % Change |
| Theme 3: *Knowledge of developmental neurotoxicity* | | | | | | | | | | |
| Question 1 | Toxic chemicals in our day-to-day lives, like air pollution or lead in drinking water, can increase a child’s risk of developing conditions like ADHD or autism. | | Collapsed Across Groups | | 34.89 | 40.67 | 5.78* | 34.40 | 43.89 | 9.49* |
|  |  |  | Country |  |  |  |  |  |  |  |
|  |  |  |  | Canada | 11.37 | 5.42 | -5.95  *** | 15.79 | 8.21 | -7.58  *** |
|  |  |  |  | Australia | 10.49 | 5.97 | -4.52 | 11.7 | 6.29 | -5.41  *** |
|  |  |  |  | India | 39.05 | 42.95 | 3.9 | 40.67 | 29.92 | -10.75  **** |
|  |  |  |  | United Kingdom | 8.78 | 7.05 | -1.73 | 8.71 | 4.95 | -3.76 |
|  |  |  |  | United States | 30.31 | 38.61 | 8.3* | 23.12 | 50.63 | 27.51** |
|  |  |  | Pregnancy Status |  |  |  |  |  |  |  |
|  |  |  |  | Yes | 14.2 | 12.91 | -1.29 | 14.45 | 7.65 | -6.8  *** |
|  |  |  |  | No | 85.8 | 87.09 | 1.29 | 85.55 | 92.35 | 6.8*s |
|  |  |  | Levels of Education |  |  |  |  |  |  |  |
|  |  |  |  | High school or less | 20.3 | 13.09 | -7.21  *** | 18.53 | 12.53 | -6  *** |
|  |  |  |  | Some college or university, no degree/  diploma | 14.76 | 15.95 | 1.19 | 14.08 | 15.48 | 1.4 |
|  |  |  |  | Bachelor's degree or diploma | 40.78 | 45.32 | 4.54 | 41.73 | 48.19 | 6.46* |
|  |  |  |  | Master's, doctorate or professional degree | 24.15 | 25.63 | 1.48 | 25.66 | 23.8 | -1.86 |
|  |  |  | Age |  |  |  |  |  |  |  |
|  |  |  |  | 18-24 years | 21.61 | 17.25 | -4.36 | 24.69 | 14.25 | -10.44  **** |
|  |  |  |  | 25-32 years | 33.86 | 32.21 | -1.65 | 32.05 | 31.77 | -0.28 |
|  |  |  |  | 33-39 years | 25.96 | 28.52 | 2.56 | 26.88 | 32.44 | 5.56* |
|  |  |  |  | 40+ years | 18.56 | 22.02 | 3.46 | 16.38 | 21.54 | 5.16* |
| Question 5 | Reducing exposure to toxic chemicals during pregnancy and in early childhood can help lower a child’s risk of developing a condition like ADHD or autism. | | Collapsed Across Groups | | 35.40 | 39.99 | 4.59 | 35.57 | 43.35 | 9.78* |
|  |  |  | Country |  |  |  |  |  |  |  |
|  |  |  |  | Canada | 13.43 | 7.15 | -6.28  *** | 16.31 | 6.43 | -9.88  *** |
|  |  |  |  | Australia | 11.94 | 7.37 | -4.57 | 12.84 | 6.84 | -6  *** |
|  |  |  |  | India | 32.59 | 37.09 | 4.5 | 33.3 | 22.57 | -10.73  **** |
|  |  |  |  | United Kingdom | 8.87 | 6.26 | -2.61 | 10.2 | 5.19 | -5.01  *** |
|  |  |  |  | United States | 33.17 | 42.12 | 8.95* | 27.35 | 58.98 | 31.63** |
|  |  |  | Pregnancy Status |  |  |  |  |  |  |  |
|  |  |  |  | Yes | 11.8 | 10.12 | -1.68 | 13.11 | 8.23 | -4.88 |
|  |  |  |  | No | 88.2 | 89.88 | 1.68 | 86.89 | 91.77 | 4.88 |
|  |  |  | Levels of Education |  |  |  |  |  |  |  |
|  |  |  |  | High school or less | 20.28 | 13.57 | -6.71  *** | 18.46 | 13.25 | -5.21  *** |
|  |  |  |  | Some college or university, no degree/  diploma | 15.13 | 14.93 | -0.2 | 14.69 | 16.31 | 1.62 |
|  |  |  |  | Bachelor's degree or diploma | 42.04 | 46.49 | 4.45 | 42.67 | 50.17 | 7.5* |
|  |  |  |  | Master's, doctorate or professional degree | 22.55 | 25 | 2.45 | 24.18 | 20.28 | -3.9 |
|  |  |  | Age |  |  |  |  |  |  |  |
|  |  |  |  | 18-24 years | 19.57 | 13.97 | -5.6  *** | 22.8 | 12.52 | -10.28  **** |
|  |  |  |  | 25-32 years | 32.59 | 31.62 | -0.97 | 32.29 | 31.88 | -0.41 |
|  |  |  |  | 33-39 years | 28.32 | 32.29 | 3.97 | 27.47 | 34.02 | 6.55* |
|  |  |  |  | 40+ years | 19.53 | 22.12 | 2.59 | 17.44 | 21.58 | 4.14 |
| Question 8 | Exposure to toxic chemicals during pregnancy can increase a child’s risk of having a developmental condition. | | Collapsed Across Groups | | 47.11 | 48.78 | 1.67 | 47.10 | 55.41 | 8.31* |
|  |  |  | Country |  |  |  |  |  |  |  |
|  |  |  |  | Canada | 14.91 | 7.47 | -7.44  *** | 17.18 | 7.01 | -10.17  **** |
|  |  |  |  | Australia | 13.44 | 8.03 | -5.41  *** | 13.23 | 7.35 | -5.88  *** |
|  |  |  |  | India | 26.95 | 31.83 | 4.88 | 27.85 | 21.29 | -6.56  *** |
|  |  |  |  | United Kingdom | 10.85 | 7.75 | -3.1 | 11.62 | 5.24 | -6.38  *** |
|  |  |  |  | United States | 33.85 | 44.93 | 11.08** | 30.12 | 59.12 | 29** |
|  |  |  | Pregnancy Status |  |  |  |  |  |  |  |
|  |  |  |  | Yes | 11.42 | 9.24 | -2.18 | 12.08 | 7.72 | -4.36 |
|  |  |  |  | No | 88.58 | 90.76 | 2.18 | 87.92 | 92.28 | 4.36 |
|  |  |  | Levels of Education |  |  |  |  |  |  |  |
|  |  |  |  | High school or less | 20.69 | 14.73 | -5.96  *** | 19.12 | 12.92 | -6.2  *** |
|  |  |  |  | Some college or university, no degree/  diploma | 16.62 | 14.82 | -1.8 | 15.64 | 16.61 | 0.97 |
|  |  |  |  | Bachelor's degree or diploma | 42.71 | 47.62 | 4.91 | 43.45 | 49.01 | 5.56* |
|  |  |  |  | Master's, doctorate or professional degree | 19.97 | 22.83 | 2.86 | 21.79 | 21.46 | -0.33 |
|  |  |  | Age |  |  |  |  |  |  |  |
|  |  |  |  | 18-24 years | 19.69 | 14.11 | -5.58  *** | 21.26 | 12.11 | -9.15  *** |
|  |  |  |  | 25-32 years | 32.51 | 32.01 | -0.5 | 33.06 | 32.52 | -0.54 |
|  |  |  |  | 33-39 years | 27.51 | 31.37 | 3.86 | 27.71 | 33.2 | 5.49* |
|  |  |  |  | 40+ years | 20.29 | 22.51 | 2.22 | 17.97 | 22.18 | 4.21 |
| Question 14 | Toxic chemicals can be detected in the blood of most pregnant women. | | Collapsed Across Groups | | 17.48 | 18.99 | 1.51 | 16.27 | 21.61 | 5.34* |
|  |  |  | Country |  |  |  |  |  |  |  |
|  |  |  |  | Canada | 12.79 | 7.86 | -4.93 | 16.6 | 8.58 | -8.02  *** |
|  |  |  |  | Australia | 12.19 | 7.86 | -4.33 | 14.95 | 6.83 | -8.12  *** |
|  |  |  |  | India | 31.67 | 37.62 | 5.95* | 33.79 | 22.24 | -11.55  **** |
|  |  |  |  | United Kingdom | 12.62 | 9.76 | -2.86 | 12.12 | 6.13 | -5.99  *** |
|  |  |  |  | United States | 30.74 | 36.9 | 6.16* | 22.53 | 56.22 | 33.69** |
|  |  |  | Pregnancy Status |  |  |  |  |  |  |  |
|  |  |  |  | Yes | 15.06 | 10.53 | -4.53 | 15.42 | 6.09 | -9.33  *** |
|  |  |  |  | No | 84.94 | 89.47 | 4.53 | 84.58 | 93.91 | 9.33* |
|  |  |  | Levels of Education |  |  |  |  |  |  |  |
|  |  |  |  | High school or less | 24.1 | 12.08 | -12.02  **** | 21.44 | 12.3 | -9.14  *** |
|  |  |  |  | Some college or university, no degree/  diploma | 16.38 | 17.87 | 1.49 | 14.7 | 18.45 | 3.75 |
|  |  |  |  | Bachelor's degree or diploma | 36.54 | 45.17 | 8.63* | 38.08 | 46.05 | 7.97* |
|  |  |  |  | Master's, doctorate or professional degree | 22.98 | 24.88 | 1.9 | 25.78 | 23.2 | -2.58 |
|  |  |  | Age |  |  |  |  |  |  |  |
|  |  |  |  | 18-24 years | 17.7 | 16.9 | -0.8 | 20.03 | 10.16 | -9.87  *** |
|  |  |  |  | 25-32 years | 35.48 | 26.43 | -9.05  *** | 32.02 | 34.15 | 2.13 |
|  |  |  |  | 33-39 years | 27.77 | 32.38 | 4.61 | 27.4 | 31.87 | 4.47 |
|  |  |  |  | 40+ years | 19.05 | 24.29 | 5.24* | 20.55 | 23.82 | 3.27 |

** difference of 5 to 9.99 percent*

*** difference of 10 percent or more*

**** difference of -5 to -9.99 percent*

***** difference of -10 percent or more*
